# Coupling of store-operated calcium entry to vasoconstriction is acid-sensing ion channel 1a dependent in pulmonary but not mesenteric arteries

**DOI:** 10.1371/journal.pone.0236288

**Published:** 2020-07-23

**Authors:** Selina M. Garcia, Lindsay M. Herbert, Benjimen R. Walker, Thomas C. Resta, Nikki L. Jernigan

**Affiliations:** Department of Cell Biology and Physiology, University of New Mexico School of Medicine, Albuquerque, New Mexico, United States of America; Indiana University School of Medicine, UNITED STATES

## Abstract

Although voltage-gated Ca^2+^ channels (VGCC) are a major Ca^2+^ entry pathway in vascular smooth muscle cells (VSMCs), several other Ca^2+^-influx mechanisms exist and play important roles in vasoreactivity. One of these is store-operated Ca^2+^ entry (SOCE), mediated by an interaction between STIM1 and Orai1. Although SOCE is an important mechanism of Ca^2+^ influx in non-excitable cells (cells that lack VGCC); there is debate regarding the contribution of SOCE to regulate VSMC contractility and the molecular components involved. Our previous data suggest acid-sensing ion channel 1a (ASIC1a) is a necessary component of SOCE and vasoconstriction in small pulmonary arteries. However, it is unclear if ASIC1a similarly contributes to SOCE and vascular reactivity in systemic arteries. Considering the established role of Orai1 in mediating SOCE in the systemic circulation, we hypothesize the involvement of ASIC1a in SOCE and resultant vasoconstriction is unique to the pulmonary circulation. To test this hypothesis, we examined the roles of Orai1 and ASIC1a in SOCE- and endothelin-1 (ET-1)-induced vasoconstriction in small pulmonary and mesenteric arteries. We found SOCE is coupled to vasoconstriction in pulmonary arteries but not mesenteric arteries. In pulmonary arteries, inhibition of ASIC1a but not Orai1 attenuated SOCE- and ET-1-induced vasoconstriction. However, neither inhibition of ASIC1a nor Orai1 altered ET-1-induced vasoconstriction in mesenteric arteries. We conclude that SOCE plays an important role in pulmonary, but not mesenteric, vascular reactivity. Furthermore, in contrast to the established role of Orai1 in SOCE in non-excitable cells, the SOCE response in pulmonary VSMCs is largely mediated by ASIC1a.

## Introduction

Vascular smooth muscle cell (VSMC) contraction and relaxation play an important role in the regulation of vascular resistance and blood pressure control. It is well established that contraction is triggered by an increase in intracellular free calcium concentration ([Ca^2+^]_*i*_) mediated by a rapid Ca^2+^ release from intracellular stores and transmembrane Ca^2+^ influx through a variety of plasma membrane ion channels, exchangers, and transporters. Although Ca^2+^ influx in VSMC is thought to be mediated primarily by L-type voltage-gated Ca^2+^ channels (VGCC), it has become increasingly clear that Ca^2+^ influx through non-selective cation channels (NSCC) plays an important role in regulating vascular tone. These include 1) receptor-operated channels (ROCs) which are regulated by agonist-receptor interaction and downstream signal transduction [1]; 2) capacitative or store-operated channels (SOCs) which are activated by depletion of intracellular Ca^2+^ stores [2, 3]; 3) mechanosensitive or stretch-activated channels (SACs) which are activated by membrane stretch [4, 5]; and 4) constitutively active cation channels which are spontaneously active (reviewed in [6]). However, the molecular identity and the functional role of these channels to mediate vasoconstriction is still a matter of debate. Complicating matters is the fact that multiple Ca^2+^-permeable channels are expressed in a given vascular bed and the functional relevance of different Ca^2+^ channels within the vascular bed remains unclear. Therefore, it is necessary to gain a better understanding of the heterogeneity that exists in Ca^2+^ signaling among vascular beds.

Increases in VSMC [Ca^2+^]_*i*_ in response to neurohumoral stimuli (norepinephrine/epinephrine) and vasoactive peptides (angiotensin II and endothelin-1) results from activation of phospholipase C (PLC) associated G-protein coupled receptors [7]. Activation of PLC leads to the hydrolysis of phosphatidylinositol 4,5-bisphosphate (PIP_2_) generating the second messengers, inositol trisphosphate (IP_3_) and diacylglycerol (DAG). IP_3_ activates IP_3_ receptors (IP_3_R) on the sarcoplasmic reticulum (SR) and stimulates Ca^2+^ release. Plasma membrane SOCs are activated secondary to depletion of SR Ca^2+^ stores, a mechanism known as store-operated Ca^2+^ entry (SOCE) or capacitative Ca^2+^ entry. SOCE facilitates the refilling of Ca^2+^-depleted SR stores and is therefore critical to SR Ca^2+^ homeostasis [8]. In VSMCs, SOCE is known to participate in other physiological processes such as vascular tone regulation [9, 10], vasculogenesis, and cell proliferation [10, 11]. However, the coupling of SOCE to contraction has been shown to differ widely among vascular beds. Snetkov et al. demonstrated that even though induction of SOCE results in similar increases in [Ca^2+^]_*i*_ in intrapulmonary, mesenteric, renal, femoral, and coronary arteries; the corresponding contraction was only observed in intrapulmonary arteries [12]. As the potential differential responses to SOCE between various vascular beds are likely due to regional differences in the molecular components of SOCE, the objective of this study is to identify the ion channels involved in coupling SOCE to vasoconstriction.

Stromal interaction molecule 1 (STIM1) is the key molecule involved in sensing levels of Ca^2+^ in the SR [13, 14]. Upon store depletion, STIM1 undergoes a conformational change, multimerizes, and translocate to regions of the SR adjacent to the plasma membrane where subsequent binding of STIM1 to Ca^2+^-permeable channels triggers the influx of Ca^2+^ across the plasma membrane [15]. Two different types of ionic current are evoked by store-depletion: 1) a high selectivity Ca^2+^ current mediated by a Ca^2+^ release-activated channel (CRAC) called Orai1 [16] and 2) NSCC current [17, 18]. Several members of the transient receptor potential canonical (TRPC) channel family have been proposed to act as SOCs (reviewed in [19, 20]), although this topic continues to be widely debated [21–23]. Previous work from our laboratory has shown that inhibition of acid-sensing ion channel 1a (ASIC1a) diminishes SOCE and associated vasoconstriction in pulmonary VSMC [24]. ASIC1a is a NSCC that belongs to the amiloride-sensitive degenerin/epithelial sodium channel (DEG/ENaC) superfamily. ASICs are known to be permeable to Na^+^, however homomeric ASIC1a channels can also conduct Ca^2+^ [25–27]. Although the ASICs are classically activated by extracellular acidosis; various non-proton ligands, effector proteins, and signaling molecules also regulate the function of ASICs [28, 29]. It is unclear if ASIC1a similarly contributes to SOCE and vascular reactivity in systemic arteries. Considering the established role of Orai1 in mediating SOCE in the systemic circulation, we hypothesize the involvement of ASIC1a in SOCE and resultant vasoconstriction is unique to the pulmonary circulation. To test this hypothesis, we have examined the roles of ASIC1a and Orai1 in SOCE- and endothelin-1-induced vasoconstriction in both small pulmonary and mesenteric arteries.

## Materials and methods

All protocols employed were reviewed and approved by the Institutional Animal Care and Use Committee of the University of New Mexico School of Medicine (Albuquerque, NM) and abide by the National Institutes of Health guidelines for animal use. Fifty-two adult male Wistar rats (200–250 g body wt, Envigo) were used in this study. Animals were housed in polyacrylic cages (1–3 per cage) supplied with bedding (shredded paper) and polycarbonate rodent tunnels and other items for environmental enrichment. Animals were housed in a specific pathogen-free animal care facility and maintained on a 12:12 hour light-dark cycle. Water and standard chow (Teklad soy protein-free diet no. 2920; Envigo) were provided *ad libitum*. Rats were anesthetized with an overdose of pentobarbital sodium (200 mg/kg ip) and immediately euthanized by exsanguination after the loss of consciousness.

### Assessment of SOCE and vasoreactivity in isolated, pressurized pulmonary and mesenteric resistance arteries

To determine simultaneous changes in vasoreactivity and [Ca^2+^]_*i*_, small resistance arteries were cannulated and pressurized for dimensional and fluorescence analysis as previously described [24, 30]. Following euthanasia, the lungs or mesentery were removed and immediately placed in PSS [pH adjusted to 7.4 with NaOH containing (in mM) 130 NaCl, 4 KC1, 1.2 MgSO_4_, 4 NaHCO_3_, 1.8 CaC1_2_, 10 HEPES, 1.18 KH_2_PO_4_, 6 glucose]. Fourth- to fifth-order pulmonary (~ 125 μm inner diameter) and third- to fourth-order mesenteric arteries (~ 150 μm inner diameter; Table 2); of ∼1-mm length and without visible side branches were dissected free and transferred to a vessel chamber (CH-1, Living Systems). The proximal end of the artery was cannulated with a tapered glass pipette, secured in place with a single strand of silk ligature, and gently flushed to remove any blood from the lumen. The vessel was stretched longitudinally to approximate in situ length and pressurized with a servo-controlled peristaltic pump (Living Systems) to 12 mmHg (pulmonary) or 75 mmHg (mesenteric). Arteries were required to hold steady pressure on switching off the servo-control function to verify the absence of leaks; any vessel with apparent leaks was discarded. The vessel chamber was superfused with PSS; at 5 ml/min at 37°C. Images were obtained using an Eclipse TS100 microscope (Nikon) and IonOptix CCD100M camera to measure inner diameter, and dimensional analysis was performed by IonOptix Ion Wizard software (IonOptix). Arteries were incubated at room temperature with PSS containing the cell-permeable ratiometric Ca^2+^-sensitive fluorescent dye fura-2 acetoxymethyl ester (fura-2 AM, 2 μM; Life Technologies, F1201, lot #2021739) and 0.02% pluronic acid (Life Technologies, P3000MP, lot #1990297) for 45 min, as previously described [31]. Fura-2-loaded vessels were alternately excited at 340 and 380 nm at a frequency of 1 Hz with an IonOptix Hyperswitch dual-excitation light source, and the respective 510-nm emissions were collected with a photomultiplier tube. After subtracting background fluorescence, emission ratios (F_340_/F_380_) were calculated with Ion Wizard software (IonOptix) and recorded continuously throughout the experiment.

Experiments were conducted in the absence or presence of the L-type VGCC inhibitor, diltiazem (50 μM; Sigma, D2521, lot #MKCD7486); the specific ASIC1a inhibitor, psalmotoxin 1 (PcTX1; 20 nM; Phoenix Peptides, 063–22, lot #433599); or the specific Orai1 inhibitor, AnCoA4 (20 μM; Millipore Sigma, 532999, lot #3030527). We have previously demonstrated that this concentration of diltiazem (50 μM) prevents increases in the vessel [Ca^2+^]_*i*_ and vasoconstriction-induced by the depolarizing stimulus, KCl (50 mM) [32]. PcTX1 has been reported to selectively inhibit ASIC1a isoform over other ASICs [33]. Furthermore, we have previously shown that this concentration of PcTX1 has similar effects to those seen in pulmonary VSMCs of ASIC1 null mice [34, 35]. AnCoA4 reduces the Orai1 association to STIM1 and consequently blocks SOCE [36]. We have previously determined this concentration of AnCoA4 inhibits SOCE in pulmonary arterial endothelial cells and pulmonary microvascular endothelial cells [37].

#### Store-operated Ca^2+^ entry

Fura-2-loaded arteries were superfused with Ca^2+^-free, PSS (in mM: 130 NaCl, 4 KC1, 1.2 MgSO4, 4 NaHCO3, 10 HEPES, 1.18 KH2PO4, 6 glucose, 3 EGTA; pH adjusted to 7.4 with NaOH) containing 50 μM diltiazem to prevent Ca^2+^ entry through L-type VGCC, and 10 μM cyclopiazonic acid (CPA; Calbiochem, 23905) to deplete intracellular Ca^2+^ stores and prevent Ca^2+^ reuptake through the sarcoplasmic reticulum Ca^2+^-ATPase for 15 minutes before replenishing the perfusate with Ca^2+^. The changes in [Ca^2+^]_*i*_ were determined upon the repletion of HEPES-based PSS containing 1.8 mM CaCl_2_ in the continued presence of diltiazem and CPA. SOCE was calculated as the change (Δ) in fura-2 ratio between Ca^2+^-depleted state and Ca^2+^-depleted state.

#### Endothelin-1 responses

Endothelin-1 (ET-1; Sigma-Aldrich, E7764, lot #088M4849V) induced vasoconstrictor reactivity and changes in the vessel wall [Ca^2+^]_*i*_ were assessed by superfusion (5 ml/min at 37°C) of cumulative concentrations of ET-1 in isolated pulmonary (10^−11^ to 10^−7^ M) and mesenteric (10^−11^ to 10^−8^ M) arteries.

### Generation of primary pulmonary and mesenteric smooth muscle cell cultures

Intrapulmonary and mesenteric arteries (~2nd-5th order) were dissected from surrounding tissue and enzymatically digested. Pulmonary arteries were digested in reduced-Ca^2+^ Hank’s balanced salt solution (HBSS) containing papain (26 U/ml), type-I collagenase (1,750 U/ml), dithiothreitol (1 mg/ml), and BSA (2 mg/ml) at 37°C for 30 min. Mesenteric arteries were digested in reduced-Ca^2+^ HBSS containing HEPES (15 mM), elastase (11.25 U/ml), soybean trypsin inhibitor type 1-S (1 mg/ml), type-I collagenase (180 U/ml), and BSA (2 mg/ml) at 37°C for 45 min. Single smooth muscle cells were dispersed by gentle trituration with a fire-polished pipette in Ca^2+^-free HBSS. Pulmonary VSMC were plated in Ham’s F-12 media supplemented with 5% fetal bovine serum and 1% penicillin/streptomycin. Mesenteric VSMC were plated in DMEM media supplemented with 10% fetal bovine serum, L-glutamine (2 mM), HEPES buffer (25 mM), and 1% penicillin/streptomycin. All cells were grown in a humidified atmosphere of 5% CO_2_-95% air at 37°C. Pulmonary and mesenteric VSMC purity was verified by morphological appearance and the presence of smooth muscle 22α (transgelin; S1 Fig). Furthermore, PCR showed the appearance of smooth muscle α-actin and lacked expression of the neuronal marker, calcitonin gene-related peptide (S1 Fig).

### Determination of ASIC1a and Orai1 expression

#### RT-PCR

Total RNA was extracted using TRIzol from pulmonary and mesenteric arteries. Brain tissue was used as a positive control. One μg of total RNA was reversed transcribed into cDNA using the Transcription First-Strand cDNA Synthesis kit (Roche). PCR was performed on cDNA with the iCycler PCR system (Bio-Rad) using REDExtract-N_Amp PCR ReadyMix (Sigma) and specific primers (Table 1) to detect transcripts for ASIC1a, Orai1, and β-actin. PCR products were separated using gel electrophoresis on a 3% agarose gel and stained with ethidium bromide for visualization under UV light.

**Table 1:** Primers and base pair (bp) product size used for RT-PCR for ASIC1a, Orai1, and β-actin.

	Primer Pair Sequence	Product Size, bp
Orai1		357 bp
Forward	5’-ACGTCCACAACCTCAACTCC-3’	
Reverse	5’-ACTGTCGGTCCGTCTTATGG-3’	
ASIC1a		305 bp
Forward	5’-GCCTATGAGATCGCAGGG-3’	
Reverse	5’-AAAGTCCTCAAACGTGCCTC-3’	
β-actin		244 bp
Forward	5’-AGTGTGACGTTGACATCCGT-3’	
Reverse	5’-GACTCATCGTACTCCTGCTT-3’	

#### Western blotting analysis

Orai1 and ASIC1a protein expression were determined by Western blot analysis. Pulmonary and mesenteric arteries were homogenized in 10 mM Tris·HCl homogenization buffer (containing 255 mM sucrose, 2 mM EDTA, 12 μM leupeptin, 1 μM pepstatin A, and 0.3μM aprotinin) with a glass homogenizer. The lysate (60 μg) was separated by SDS-PAGE (7.5% or 12% Tris/glycine) and transferred to a polyvinylidene difluoride membrane. The blot was blocked for 1 h with 5% milk and then incubated at 4°C with rabbit anti-Orai1 (1.5 hrs @ 1:300; Proteintech: 14443-1-AP; expected MW 35–44 kDa) or rabbit anti-ASIC1a (48 hrs @ 1:500; Millipore: AB5674P; reported MW ~60 and ~100 kDa). For immunochemical labeling, blots were incubated with anti-rabbit IgG-horseradish peroxidase (1 hr @ 1:3,000; Bio-Rad). Following chemiluminescence labeling (ECL; Pierce), Proteins were detected by exposing the blot to chemiluminescence-sensitive film (GeneMate).

### Determination of ASIC1a-STIM1 and Orai1-STIM1 colocalization

Protein-protein interactions were determined in smooth muscle cells using Duolink in situ proximity ligation assay (PLA) as previously described [34, 38]. Cells were plated on 18-well slides (Ibidi) and grown until ~75% confluent. In some experiments, cells were pretreated with Ca^2+^ free PSS plus CPA to induce SOCE before the cells were fixed with 2% paraformaldehyde. Following fixation, samples were incubated with Duolink blocking buffer for 30 min at 37°C then incubated overnight with mouse anti-STIM1 (1:50; BD Biosciences 610954) and goat anti-ASIC1a (1:50; Santa Cruz Biotechnology: sc-13903) or rabbit anti-Orai1 (1:100; Alomone ACC-062). We have previously determined the specificity of goat anti-ASIC1a using wild-type and knockout mice [39], and the specificity of rabbit anti-Orai1 using control and siRNA-treated cells [37]. Cells were then incubated with anti-mouse PLUS and anti-goat MINUS or anti-rabbit MINUS PLA probes (1:5) for 1 h at 37°C. Negative controls were completed by incubation of each primary antibody individually. Samples were amplified with Duolink In Situ Detection Reagent Orange (excitation/emission: 554/579 nm; Sigma- Aldrich) for 100 min at 37°C. SYTOX Green (1:10,000; Invitrogen) was used as a nuclear stain and actin was stained with Alexa Fluor 647 Phalloidin (1:100; Invitrogen). Samples were mounted with Duolink mounting media and Z-stack images of the PLA interaction were acquired using a confocal microscope (TCS SP5; Leica). Each puncta was considered a positive protein-protein interaction. The number and size (pixel^2^) of puncta per cell were determined using ImageJ (National Institutes of Health).

### Calculations and statistics

All data are expressed as means ± SE. Values of n refer to the number of animals in each group unless otherwise stated. Statistical significance was tested at the 95% (P < 0.05) confidence level using an unpaired t-test, one-way analysis of variance (ANOVA), or two-way ANOVA as appropriate (GraphPad Prism). If differences were detected by ANOVA, individual groups were compared with the recommended post-hoc test which is specified in the figure legends. Normal distribution was tested using the Shapiro-Wilks Normality Test (P > 0.05), any data sets that were not normally distributed were analyzed by non-parametric analysis. Data that were represented as percent were normalized by arcsine transformation before statistical analysis.

## Results

### L-type Ca^2+^ channels contribute to ET-1-induced constriction of mesenteric but not pulmonary arteries

**Fig 1:**
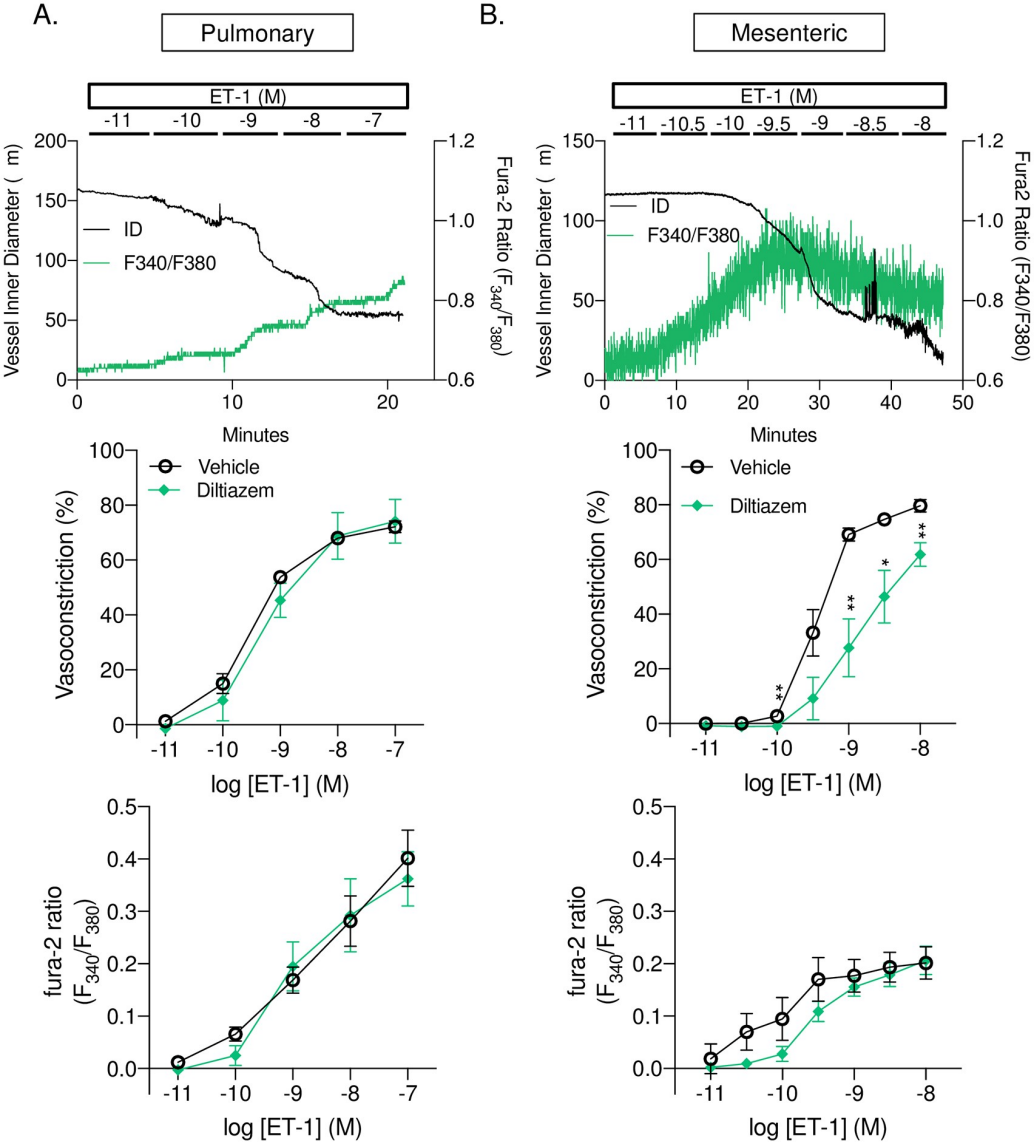
L-type Ca^2+^ channels contribute to ET-1 induced vasoconstriction in mesenteric but not pulmonary arteries. Representative traces (top panels) and summary data showing vasoconstriction (percent baseline inner diameter; middle panels) and changes in fura-2 ratio (F_340_/F_380_; bottom panels) in response to endothelin-1 (ET-1; 10^−11^–10^−7^ or 10^−11^–10^−8^) in the presence or absence of diltiazem (50 μM) in small pulmonary (A; left panels) or mesenteric (B; right panels) arteries. n = 4–6 per group, values are means ± SEM. *p ≤ 0.05 vs. vehicle; ** p < 0.01; analyzed by two-way ANOVA followed by Sidak’s multiple comparisons test.

Since L-type Ca^2+^ channels are considered to be a major contributor to VSMC Ca^2+^ influx, we first determined the role of L-type VGCC to ET-1 induced vasoconstriction in small pulmonary and mesenteric arteries. In pulmonary arteries, inhibition of L-type VGCC with diltiazem did not significantly alter baseline diameter or vessel wall [Ca^2+^]_*i*_ (Table 2). Furthermore, diltiazem did not affect vasoconstriction (Fig 1A, top panel) or changes in vessel wall Ca^2+^ (Fig 1A, bottom panel) in response to increasing doses of ET-1. In mesenteric arteries, inhibition of L-type VGCC with diltiazem did not significantly alter baseline diameter or vessel wall [Ca^2+^]_*I*_ (Table 2). However, ET-1-induced vasoconstriction in mesenteric arteries was significantly attenuated by diltiazem (Fig 1B, top panel) and tended to decrease changes in vessel wall Ca^2+^ but was not statistically significant (Fig 1B, bottom panel).

**Table 2:** Baseline inner diameter and vessel wall [Ca^2+^]_*i*_ in pulmonary and mesenteric arteries treated with vehicle, diltiazem (50 μM), PcTX1 (20 nM), or AnCoA4 (20 μM).

	Pulmonary					Mesenteric				
	Diameter	P-Value	Fura-2 ratio (F_340_/F_380_)	P-Value	N	Diameter	P-Value	Fura-2 ratio (F_340_/F_380_)	P-Value	N
	(μm)					(μm)				
**Vehicle**	124 ± 7		0.79 ± 0.06		7	149 ± 15		0.66 ± 0.06		6
**Diltiazem**	125 ± 19	>0.9999	0.61 ± 0.11	0.9665	4	165 ± 12	0.8575	0.64 ± 0.02	0.9823	6
**PcTX1**	119 ± 9	0.9763	0.82 ± 0.06	0.9699	7	166 ± 11	0.8648	0.75 ± 0.03	0.5156	5
**AnCoA4**	122 ± 11	0.9982	0.78 ± 0.06	0.9999	7	139 ± 17	0.9535	0.59 ± 0.05	0.6305	7

### Orai1 and ASIC1a mRNA and protein expression in pulmonary and mesenteric arteries

**Fig 2:**
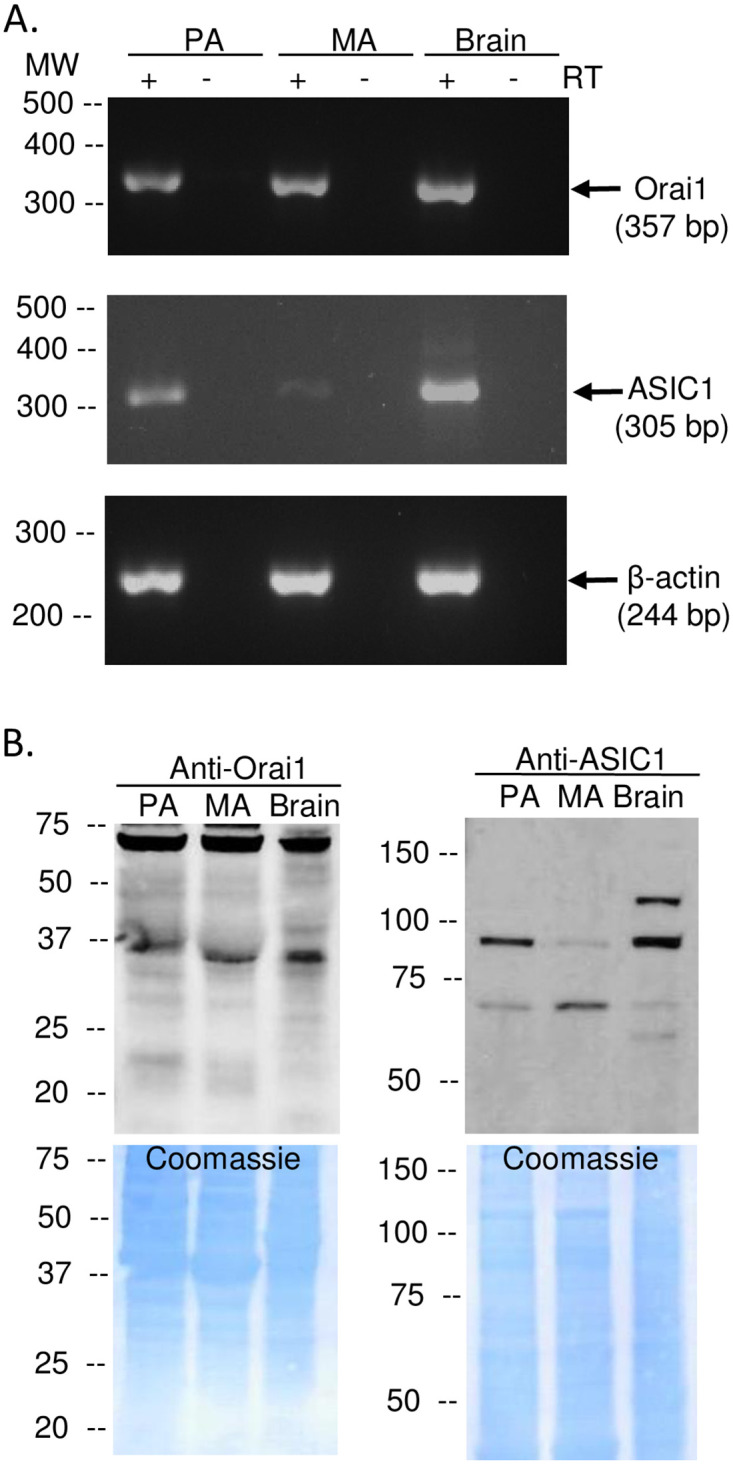
Orai1 and ASIC1a are expressed in pulmonary and mesenteric arteries and VSMC. Representative PCR gels (A) showing expression of Orai1 (357 bp) and ASIC1a (305 bp) in intact pulmonary arteries (PA), mesenteric arteries (MA), and brain tissue (positive control). All lanes were loaded with 5 μL cDNA. β-actin (244 bp) was used as a loading control for intact arteries. Representative western blots (B left) showing protein expression of Orai1 and ASIC1 (~100 kDa and 60 kDa) in intact PA, MA, and brain tissue (positive control). Coomassie blue staining shows equal protein loading between samples (B; right). Each experiment was replicated 3 times.

We next evaluated mRNA and protein expression of Orai1 and ASIC1 in small pulmonary and mesenteric arteries. We found Orai1 and ASIC1 transcripts (Fig 2A) and protein (Fig 2B) were expressed in both pulmonary and mesenteric arteries. Brain tissue was used as a positive control. Orai1 was detected around the expected MW of 35 kDa and a larger band around 75 kDa (a potential dimer). Similar to previous reports [40], we detect two bands for ASIC1 protein, ~100 kDa and 60 kDa. Interestingly, the 60 kDa band is more intense in mesenteric arteries compared to pulmonary arteries or brain samples.

### Store-operated Ca^2+^ entry is coupled to vasoconstriction in pulmonary but not mesenteric arteries

**Fig 3:**
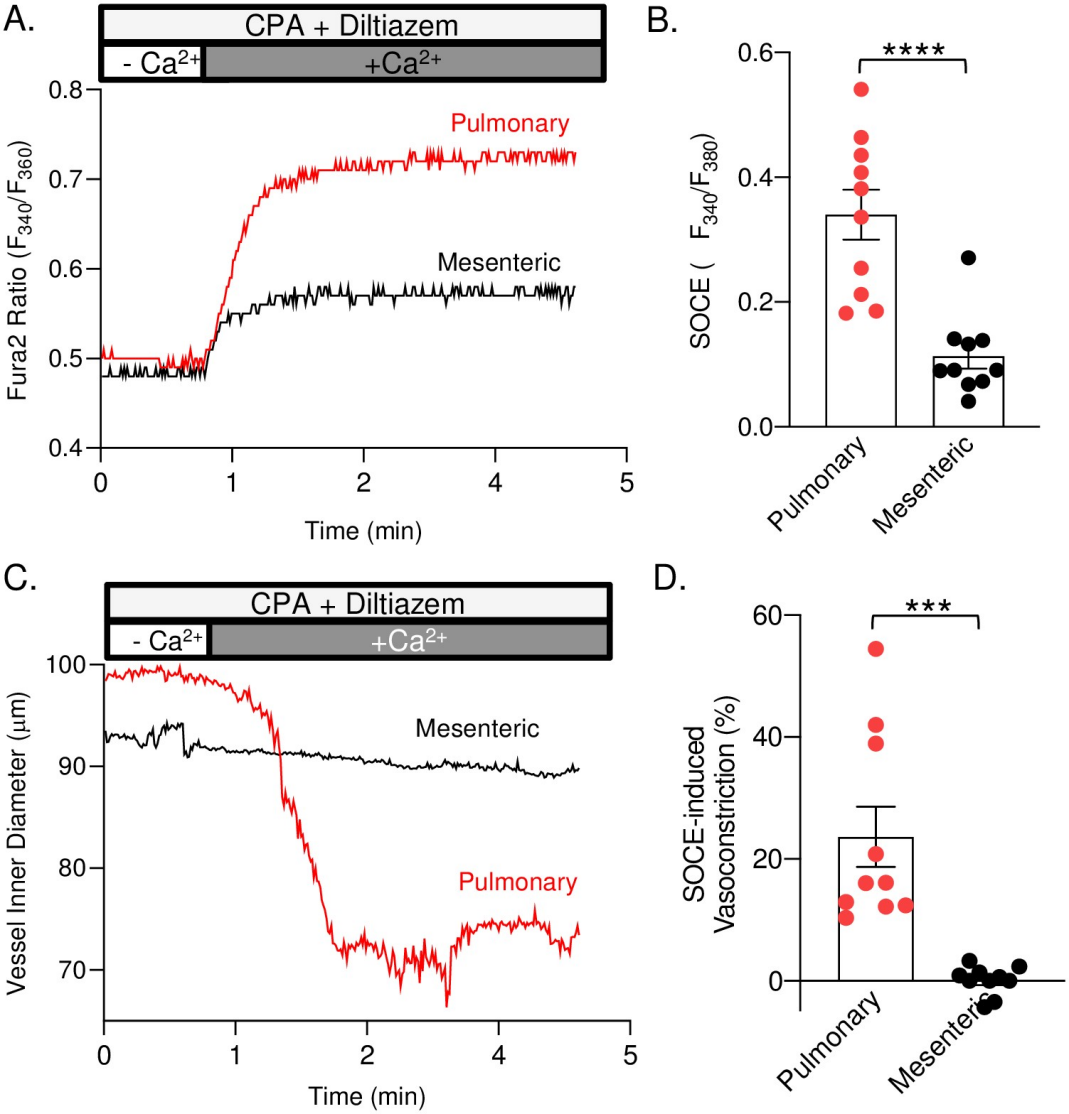
Store-operated Ca^2+^ entry is coupled to vasoconstriction in pulmonary but not mesenteric arteries. Representative traces and summary data of SOCE (A & B) and SOCE-induced vasoconstriction (C & D) in pulmonary and mesenteric resistance arteries. All studies were done in the presence of diltiazem (50μM) and cyclopiazonic acid (10μM). n = 7–10 animals/group. ***p ≤ 0.001 or **** p ≤ 0.0001 vs. pulmonary; analyzed by unpaired t-test.

Induction of SOCE in isolated pressurized small pulmonary arteries resulted in a substantial and sustained increase in [Ca^2+^]_*i*_ (Fig 3A and 3B) that was associated with an ~25% vasoconstriction (Fig 3C and 3D). In mesenteric arteries, induction of SOCE also resulted in a sustained increase in [Ca^2+^]_*i*_. However, SOCE was significantly less compared to pulmonary arteries (Fig 3A and 3B). Furthermore, SOCE was not associated with a significant change in vessel inner diameter in mesenteric arteries (Fig 3C and 3D).

### Store-operated Ca^2+^ entry is dependent on ASIC1a in pulmonary but not mesenteric arteries

**Fig 4:**
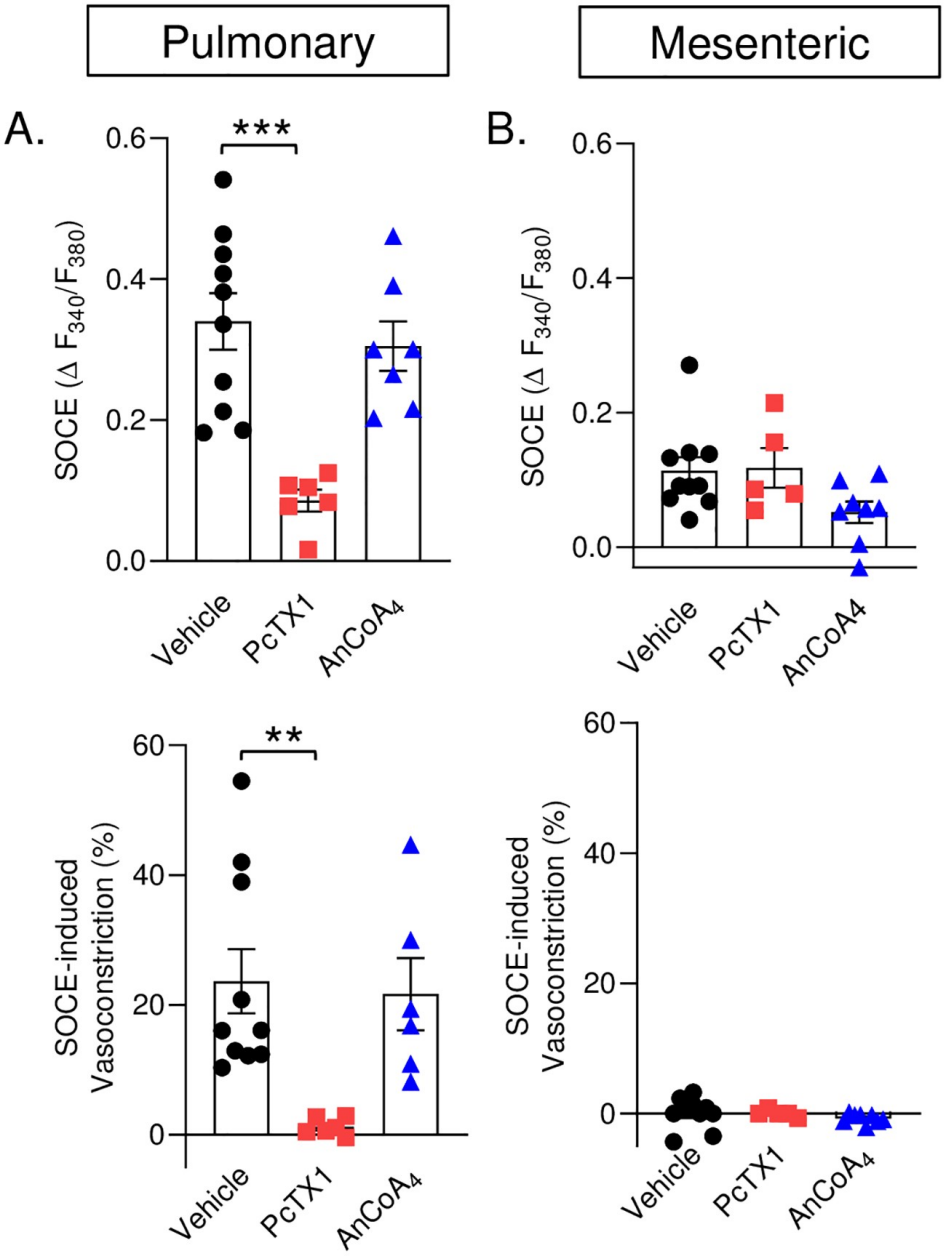
Store-operated Ca^2+^ entry is dependent on ASIC1a in pulmonary but not mesenteric arteries. SOCE-induced changes in fura-2 ratio (Δ F_340_/F_380_; top panels) and associated vasoconstriction (% baseline; bottom panels) in small pulmonary and mesenteric arteries, respectively. Studies were conducted in the presence or absence of PcTX1 (20 nM) or AnCoA_4_ (20 μM). Vehicle data are the same as Fig 3. n = 5–10 animals/group. **p ≤ 0.01 vs. vehicle; *** p ≤ 0.001 vs. vehicle; analyzed by one-way ANOVA and individual groups compared with the Tukey’s multiple comparison test.

To determine the contribution of Orai1 and ASIC1a to SOCE in small pulmonary and mesenteric arteries, we repeated experiments in the presence of the ASIC1a inhibitor, PcTX1; or the Orai1 inhibitor, AnCoA4. Inhibition of ASIC1a largely attenuated the SOCE and associated vasoconstrictor responses in pulmonary arteries; however, Orai1 inhibition did not alter responses to store depletion compared to the vehicle (Fig 4A). In contrast, we observed a minimal SOCE response, in mesenteric arteries, which did not induce vasoconstriction. SOCE was not altered by PcTX1 in mesenteric arteries. Although AnCoA4 tended to blunt SOCE in mesenteric arteries, this effect was not significant (P = 0.0717). Neither PcTX1 nor AnCoA4 affected the lack of vasoconstriction in mesenteric arteries. However, the power of statistical comparison is low (0.435) in mesenteric arteries and may be due to the small effect size that we see in the mesenteric SOCE response. Together, these data demonstrate SOCE in mesenteric arteries is a negligible Ca^2+^ influx pathway compared to pulmonary arteries.

### ASIC1a and Orai1 interact with STIM1 in VSMC

**Fig 5:**
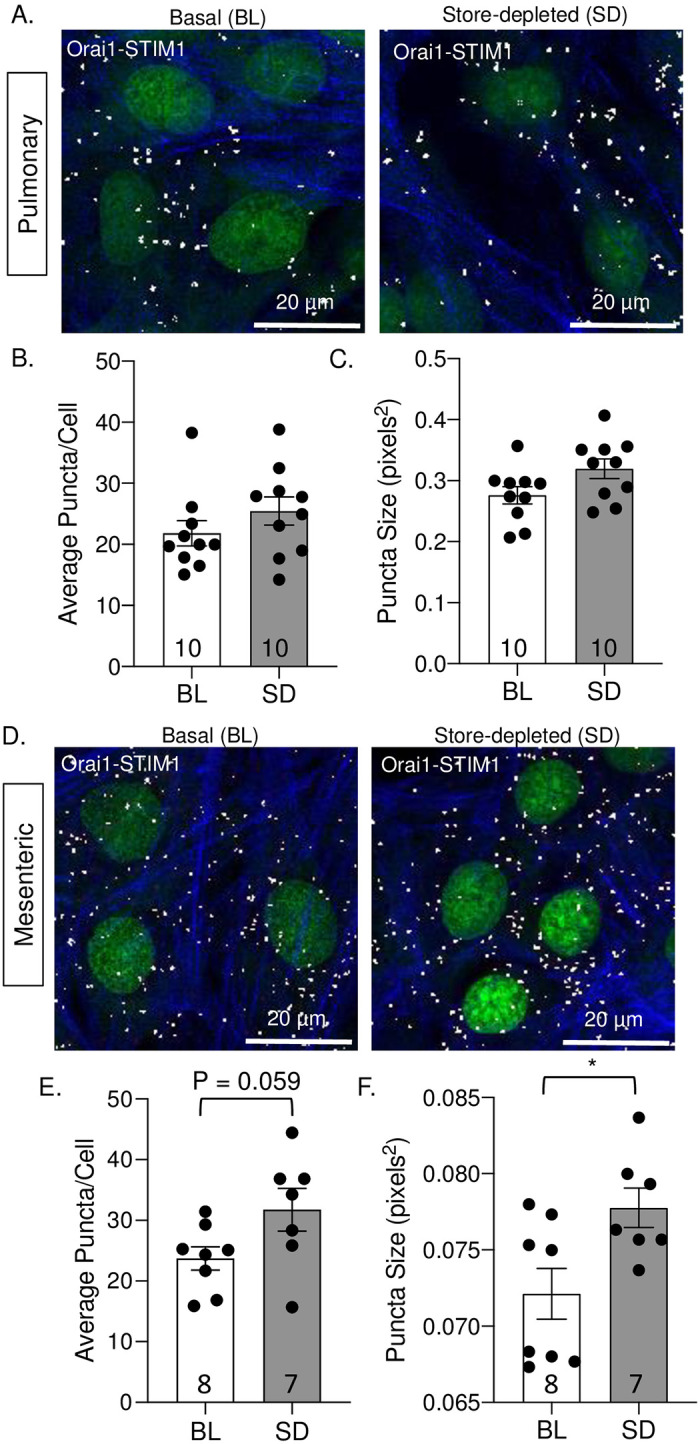
Store depletion increases the interaction between Orai1 and STIM1 in VSMCs. Representative images of Orai1/STIM1 interaction in pulmonary (A) and mesenteric (D) VSMCs under basal conditions (BL; left) or following store-depletion (SD; right). Summary data showing the average number of puncta per cell (B and E) and puncta size (C and F) in pulmonary (B-C) and mesenteric (E-F) VSMCs. The nuclei are labeled with SYTOX (green), actin is labeled with Alexa Fluor 647 Phalloidin (blue), and puncta formation (white). N = 7–10; each data point represents an individual well taken from 1–3 experiments (cell cultures generated from different animals and ran on different days). *P ≤ 0.05 vs. baseline.

As Ca^2+^ levels in the SR decrease, STIM1 undergoes a conformational change, multimerizes, and translocates to regions of the SR adjacent to the plasma membrane where subsequent binding of STIM1 to Ca^2+^-permeable channels triggers an influx of Ca^2+^ across the plasma membrane [15]. Thus we first examined the mRNA and protein levels of STIM1 in pulmonary and mesenteric arteries and found that STIM1 was expressed in both vascular beds (S2 Fig). Furthermore, an examination of STIM1, Orai1, and ASIC1 mRNA showed expression in both primary cultured pulmonary and mesenteric VSMCs (S3 Fig). Therefore, we examined the potential interaction and clustering of STIM1 with both Orai1 and ASIC1a in pulmonary and mesenteric VSMCs using Duolink proximity ligation assay. Under basal conditions, we found that Orai1 interacts with STIM1 in both pulmonary (Fig 5A and 5C) and mesenteric (Fig 5D and 5F) VSMCs. Upon store depletion with CPA in pulmonary VSMC, there was neither a difference in the number of puncta (interactions) per cell (Fig 5B) nor puncta size (Fig 5C). In mesenteric VSMC there tended to be both an increase in the number of puncta per cell (p = 0.0593) and a significant increase in puncta size, suggesting an increase in Orai1-STIM1 clustering. However, the size of the puncta is substantially smaller than the puncta measured in pulmonary arteries. The changes observed in mesenteric VSMC may be due to possible phenotypic changes that can occur in VSMs in cell culture from a quiescent to the proliferative state. Furthermore, the power of statistical comparison was lower than expected when analyzing the number of puncta per cell in mesenteric VSMCs (0.623).

**Fig 6:**
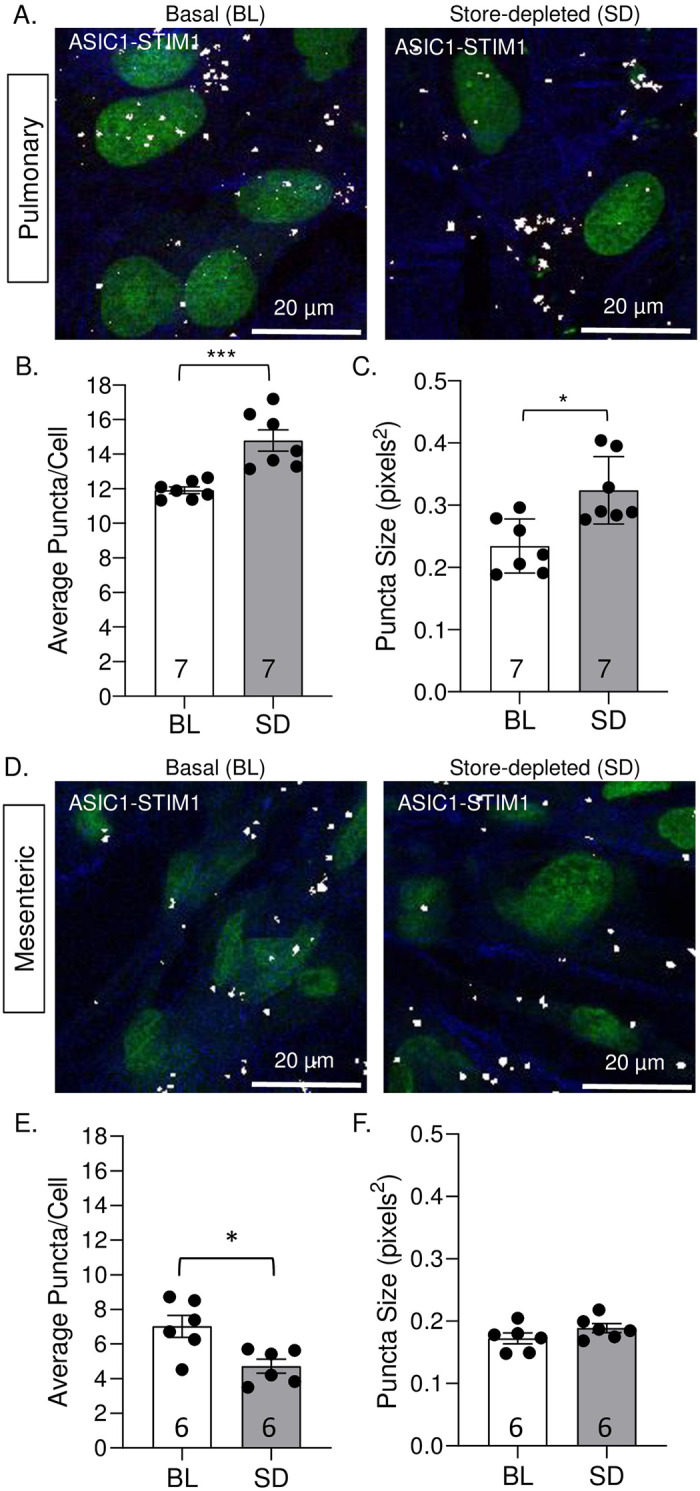
Store depletion increases the interaction between ASIC1a and STIM1 in pulmonary, but not in mesenteric VSMCs. Representative images of ASIC1a/STIM1 interaction in pulmonary (A) and mesenteric (D) VSMCs under basal conditions (BL; left) and following store-depletion (SD; right). Summary data showing the average number of puncta per cell (B and E) and puncta size (C and F) in pulmonary (B-C) and mesenteric (E-F) VSMCs. The nuclei are labeled with SYTOX (green), actin is labeled with Alexa Fluor 647 Phalloidin (blue), and puncta formation (white). n = 6–7 wells; each data point represents an individual well taken from 1–3 experiments (cell cultures generated from different animals and ran on different days). *P < 0.05 vs. baseline; **P < 0.01 vs. baseline; *** p < 0.001 vs. baseline; analyzed by unpaired t-test, except for C which was analyzed using Mann-Whitney test.

ASIC1a also interacts with STIM1 under basal conditions in both pulmonary (Fig 6A) and mesenteric (Fig 6D) VSMCs. Upon store depletion in pulmonary VSMC there was a significant increase in the number of puncta (interactions) per cell (Fig 6B) and a significant increase in puncta size (Fig 6C), indicating clustering of ASIC1a and STIM1 proteins. Compared to pulmonary VSMC, there were significantly fewer puncta/cell in mesenteric VSMC. In addition, store depletion in mesenteric VSMC resulted in a significant decrease in the number of puncta (interactions), per cell (Fig 6E) but no change in puncta size (Fig 6F).

In negative control experiments, we incubated each primary antibody individually and observed no positive interactions (S4 Fig). Taken together, these data demonstrate a physical interaction between ASIC1a and STIM1 in pulmonary VSMCs and support our findings that ASIC1a functions as a SOC in the pulmonary circulation.

### Vasoconstrictor and arterial wall [Ca^2+^]_*i*_ responses to ET-1 are attenuated in response to ASIC1a inhibition and augmented in response to Orai1 inhibition in pulmonary arteries

**Fig 7:**
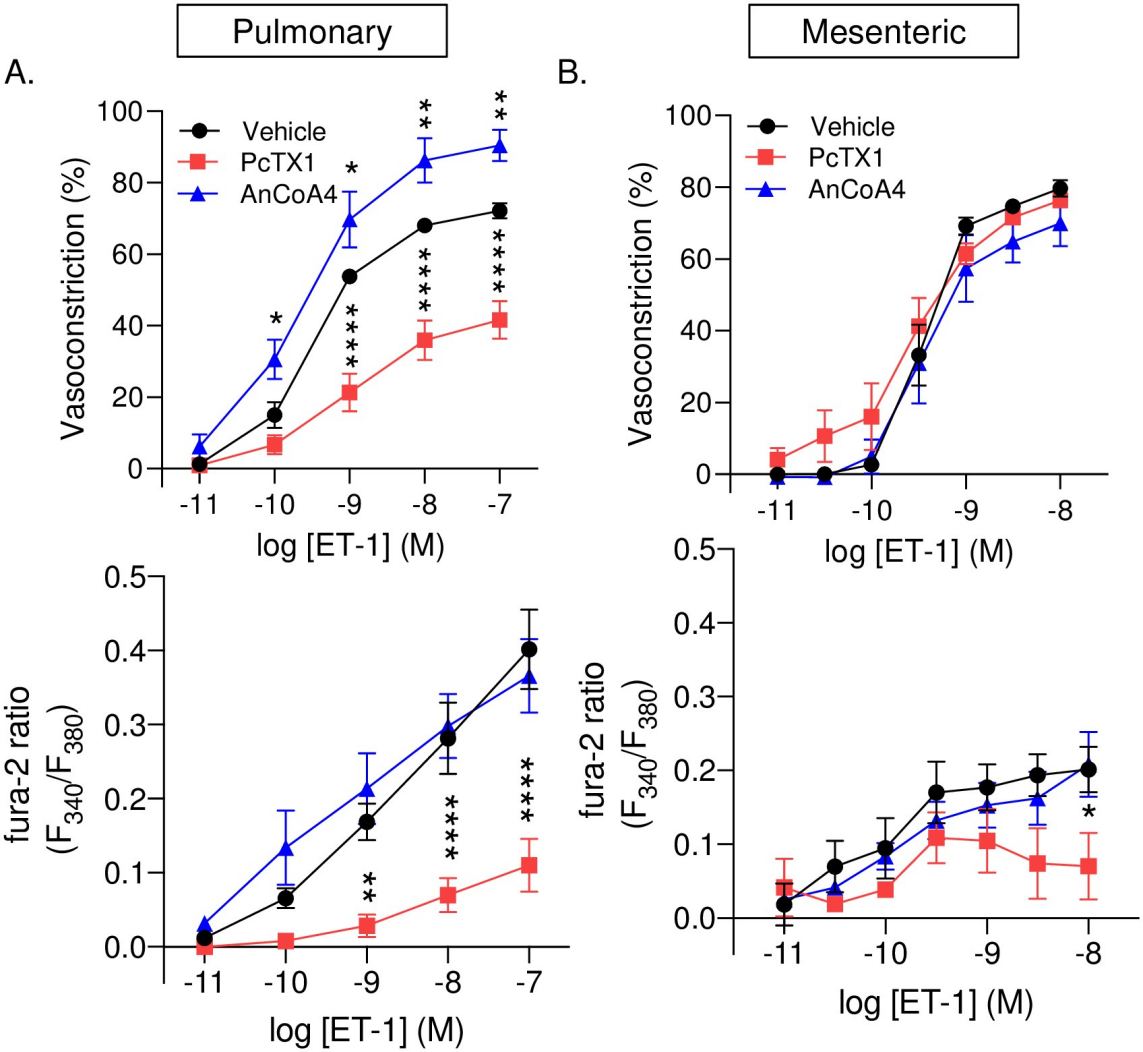
Vasoconstrictor and arterial wall [Ca^2+^]_i_ responses to ET-1 are attenuated in response to ASIC1a inhibition and augmented in response to Orai1 inhibition in pulmonary arteries. Vasoconstriction (percent baseline inner diameter; top panels) and changes in fura-2 ratio (F_340_/F_380_; bottom panels) in response to endothelin-1 (ET-1; 10^−11^–10^−7^ or 10^−11^–10^−8^) in the presence or absence of PcTX1 (20 nM) or AnCoA_4_ (20 μM) in small pulmonary (A) and mesenteric (B) arteries. N = 4–7 per group, values are means ± SEM. *p ≤ 0.05 vs. vehicle; ** p < 0.01, *** p ≤ 0.001, **** p ≤ 0.0001; analyzed by two-way anova followed by Dunnett’s multiple comparisons test.

Orai1 channels have also been shown to play a vasoactive role that is independent of SOCE [41]. It is possible that both ASIC1a and Orai1 participate in ligand-activated Ca^2+^ entry in VSMCs and vasoconstriction through a store-independent mechanism; therefore, we further assessed the role of ASIC1a and Orai1 in ET-1-induced vasoconstriction. In both pulmonary and mesenteric arteries, inhibition of ASIC1a or Orai1 did not significantly alter baseline diameter or vessel wall [Ca^2+^]_*i*_ (Table 2). Consistent with previous studies showing ASIC1a contributes to agonist-induced vasoreactivity in small pulmonary arteries [31, 39, 42], PcTX1 reduced ET-1-mediated vasoconstriction and arterial wall [Ca^2+^]_*i*_ responses in small pulmonary arteries (Fig 7A). In contrast, Orai1 inhibition with AnCoA4 enhanced ET-1-induced vasoconstriction but did not alter [Ca^2+^]_*i*_ responses in pulmonary arteries (Fig 7A). Interestingly, in mesenteric arteries we found that PcTX1 did not affect ET-1-induced vasoconstriction but significantly attenuated arterial wall [Ca^2+^]_*i*_ responses in small mesenteric arteries at the highest concentration of ET-1 (Fig 7B). Further examination with AnCoA4 indicated no change in vasoconstriction or arterial wall [Ca^2+^]_*i*_ in response to ET-1 (Fig 7B). Together, these studies suggest that ASIC1a and Orai1 play unique roles in pulmonary but not mesenteric artery constriction.

## Discussion

The goal of this study was to compare the contribution of SOCE to vasoconstriction and potential mediators of this response between small pulmonary and mesenteric arteries. The major findings of this study are that 1) the contribution of L-type VGCC to agonist-induced vasoconstriction in pulmonary and mesenteric arteries is minimal; 2) SOCE is functionally linked to vasoconstriction in pulmonary but not mesenteric arteries; and 3) ASIC1a is the major contributor of SOCE and SOCE-induced vasoconstriction in pulmonary arteries; while Orai1 plays a minimal role in vasoconstriction in either bed. Together, these data demonstrate a unique role for ASIC1a in the pulmonary circulation where it contributes to both SOCE-induced and agonist-induced vasoconstriction; yet ASIC1a does not contribute to vasoconstriction in the mesenteric circulation.

A variety of Ca^2+^ influx pathways have been identified in VSMC, including (but not limited to) VGCC, SOC, and ROC. The contribution of these various Ca^2+^ influx pathways utilized by VSMC to elicit contraction varies widely across different vascular beds. Therefore, we first investigated the role of L-type VGCC in both pulmonary and mesenteric agonist-induced vasoconstriction, using the antagonist diltiazem. Similar to other reports [31], there was little effect of diltiazem on agonist-induced vasoconstriction of pulmonary arteries, suggesting little role for Ca^2+^-influx through L-type VGCC in ET-1-induced vasoconstriction. Although diltiazem significantly reduced ET-1 induced vasoconstriction in mesenteric arteries, a large proportion of the vasoconstrictor response remained along with the increased [Ca^2+^]_*i*_. These findings are consistent with studies by Kawanabe et al. [43, 44] in rabbit basilar and internal carotid arteries showing a large proportion of ET-1-induced contraction is L-type VGCC independent. Although there is likely a component of T-type VGCC to ET-1 increases in Ca^2+^ influx, general inhibition of all VGCC does not abolish the inward calcium currents caused by ET-1 [45]. Together, these data suggest the increases in [Ca^2+^]_*i*_ and vasoconstriction in response to ET-1 are mediated by a variety of sources.

Although SOCE has been demonstrated in several VSMC preparations, the relationship between Ca^2+^ entry via this pathway and contraction remains unclear. Contraction due to SOCE has been demonstrated in the aorta, intrapulmonary, cerebral, mesenteric and femoral arteries [12, 46–48]; while others report that elevated [Ca^2+^]_*i*_ due to SOCE appears to be dissociated from contraction in the aorta, coronary, renal, and mesenteric arteries [12, 49]. Although Snetkov et al., reported that SOCE resulted in a similar increase in Ca^2+^ between pulmonary and mesenteric arteries, regardless of coupling to contraction [12], we found the SOCE response to be significantly less in mesenteric compared to pulmonary arteries. Consequently, it is possible that the smaller SOCE response in mesenteric arteries is not sufficient to induce vasoconstriction. Interestingly, this smaller SOCE response in mesenteric arteries seems to be associated with lower ASIC1 mRNA and protein expression in mesenteric compared to pulmonary arteries, although these were not statistically compared. The failure of SOCE to elicit mesenteric vasoconstriction is supported by the observations that ASIC1a and Orai1 inhibition did not affect ET-1-induced vasoconstriction. These data suggest the component of ET-1-induced vasoconstriction that is VGCC-independent is not mediated by SOCE but rather other Ca^2+^ signaling pathways. ROCs such as TRP channels or other non-selective cation channels may play a role in ET-1-induced Ca^2+^ in mesenteric arteries. Furthermore, cytosolic Ca^2+^ levels may be altered due to the production and signaling of IP_3_ to activate IP_3_ receptors and release Ca^2+^ from Ca^2+^ stores. It is also possible that Ca^2+^ sensitization is playing a role in ET-1-induced vasoconstriction. Specifically, Ca^2+^ sensitization occurs when vasoconstriction is regulated by increasing phosphorylation of the myosin regulatory light chain independent of changes in [Ca^2+^]_i_ [50]. Therefore, it is possible that the ET-1-induced vasoconstrictor responses we see are due to ET-1-stimulated Ca^2+^ sensitization.

Expression of Orai1 was similar between pulmonary and mesenteric arteries; and although inhibition of Orai1 tended to diminish SOCE in mesenteric arteries, it did not affect pulmonary SOCE. Furthermore, inhibition of Orai1 did not affect vasoconstrictor responses in mesenteric vessels. This is in contrast to other studies showing that silencing of Orai1 or its functional inhibition with monoclonal antibodies diminishes SOCE and agonists-induced contraction in coronary and aortic rings [10, 47]. These studies suggest that in some vascular beds, Orai1 is present and functions as a SOC. Although the role of Orai1 in VSMC has been studied in several cultured VSMC preparations, it is important to note that the above studies ([10, 47]; as well as Figs 5 & 6) were conducted in freshly isolated tissue as opposed to cultured cells. Indeed, it has been shown that expression of Orai isoforms is relatively low in contractile VSMCs; however, expression of Orai1 and STIM1 dramatically increase along with SOCE in cultured VSMCs [51, 52]. VSMCs in culture are known to undergo a phenotypic switch to a synthetic or proliferative state, which often correlates with a more prominent SOCE response [53, 54]. This may explain why we observed increased clustering (indicated by increased puncta size) between Orai1 and STIM1 in response to store depletion in transiently cultured mesenteric VSMC even though Orai1 did not significantly contribute to SOCE responses in freshly isolated tissue. Together these data suggest there may be a minimal contribution of vascular Orai1-dependent SOCE to normal vascular homeostasis; however, SOCE may become a more important mechanism of Ca^2+^ influx in cardiovascular diseases involving VSMC dysfunction leading to a more proliferative, synthetic state of the VSMC [46, 55].

Although inhibition of Orai1 did not significantly alter SOCE in pulmonary arteries, it unexpectedly augmented the vasoconstrictor response in these endothelium-intact vessels. Receptors for ET-1 are expressed on both VSMC (ET_A_ and ET_B_), where activation results in vasoconstriction, and endothelial cells (ET_B_), which mediate vasodilation. Since we have previously shown an important role of Orai1 in SOCE response in pulmonary arterial endothelial cells and pulmonary microvascular endothelial cells [37], it is likely AnCoA4 inhibited endothelial Orai1 and thus ET-1 vasodilatory responsiveness leading to augmented ET-1 vasoconstriction. We did not observe a corresponding increase in the vessel wall [Ca^2+^]_*i*_, however, this is likely a result of Ca^2+^ sensitization of the contractile apparatus that occurs in pulmonary arteries [56]. It is possible that within the pulmonary circulation, ASIC1a plays a larger role in VSMC SOCE while Orai1 mediates endothelial SOCE. This is consistent with the idea that in non-excitable cells whole-cell currents activated by Ca^2+^ store depletion are predominantly mediated by Ca^2+^ release-activated Ca^2+^ current (I_CRAC_) through Orai1. Whereas, in excitable muscle cells there is a greater contribution of Ca^2+^-permeable NSCC with a linear current-voltage relationship that contributes more to the SOC current (I_SOC_) (reviewed in [57]). Although it remains controversial, transient receptor potential (TRP) channels have long been proposed as SOC candidates [22, 58].

Numerous TRP channels contribute to the regulation of membrane potential and vascular tone. Of the various subtypes, it is the canonical (TRPC) subfamily, particularly TRPC1 that has been proposed as a SOC. Several studies using siRNA knockdown or neutralizing antibodies towards TRPC1 show incomplete inhibition of SOCE in various VSMCs including the aorta, pulmonary, cerebral, and portal vein [59–62]. In addition to minimal inhibition of SOCE by targeting TRPC1, these studies were also completed in cultured cells which may alter the SOCE response. Interestingly, Dietrich et al. [63] demonstrated VSMCs of TRPC1^-/-^ mice freshly isolated from aortas and cerebral arteries showed no difference in SOCE induced by thapsigargin, IP_3_, or cyclopiazonic acid compared to cells from wild-type mice. Further investigation into the role of TRPC1 in SOCE found that TRPC1 only contributed to SOCE when both STIM1 and Orai1 were present, suggesting that TRPC1 is not be directly activated by store depletion, but rather is activated secondarily to STIM1 and Orai1 [64–66]. Therefore, although VSMC TRPC channels play an important role in vascular homeostasis, it is unlikely through SOCE.

In contrast to TRPC1^-/-^ mice where there was no observable change in VSMC SOCE response [63], ASIC1a^-/-^ mice have significantly reduced SOCE in pulmonary VSMCs [34, 38, 39]. Furthermore, transfection of the ASIC1a gene into pulmonary VSMCs from ASIC1a^−/−^ mice restores SOCE to a level similar to that in wildtypes, further demonstrating ASIC1a, *per se*, is an essential component of SOCE [39]. ASIC1a conducts both Na^+^ and Ca^2+^ [25–27], and it is possible ASIC1a-mediated Na^+^ influx leads to VSMC depolarization and secondary activation of VGCC. However, this effect is unlikely in the present study since all SOCE responses were performed in the presence of diltiazem. Further, diltiazem and PcTX1 had very different effects on ET-1-induced vasoconstriction, suggesting different mechanisms are involved. Consistent with the possibility that ASIC1a functions as a SOC in pulmonary VSMC, we found an increased interaction between ASIC1a and STIM1 in pulmonary VSMC that was not present in mesenteric VSMCs in response to store depletion. Furthermore, we observed an increase in the ASIC1a-STIM1 association upon store depletion in pulmonary VSMCs. Although we observed an association between ASIC1a and STIM1 in mesenteric VSMC, we did not measure any increase after store depletion. Therefore, suggesting that ASIC1a is not involved in SOCE in mesenteric VSMCs. It is possible that the basal interaction that we observe between ASIC1a and STIM1 in mesenteric VSMC is due to the effects of cell culture where the cells can undergo phenotypic switches possibly leading to enhanced expression of ASIC1a and STIM1 in the cells. Although we did not investigate the role of ASIC1a to SOCE in other systemic vascular beds beyond the mesenteric circulation, these studies suggest ASIC1a may have a pulmonary-specific role. Indeed, we found ASIC1a^-/-^ mice are protected from the development of chronic hypoxia-induced pulmonary hypertension [39]. More specifically, in response to chronic hypoxia, ASIC1a^-/-^ mice showed no increase in the SOCE response, vasoconstrictor reactivity, vascular remodeling, right ventricular systolic pressure or right ventricular hypertrophy observed in wildtype mice [39], suggesting a unique role for ASIC1a in development of pulmonary hypertension.

In addition to pulmonary hypertension, SOCE dysregulation is associated with several vascular disorders including atherosclerosis, systemic hypertension, and restenosis. In STIM1 smooth muscle and endothelial cell-specific knockout animals, basal systolic blood pressures were similar to those of wild-type animals [67]. However, angiotensin-II-induced hypertension was significantly attenuated in the smooth muscle-specific STIM1^-/-^ compared to wildtype animals [68], suggesting that STIM1 within the smooth muscle is a critical player in the role of angiotensin-II-induced hypertension. Similarly, the mRNA and protein levels of STIM1 and Orai1 were significantly higher and there was a significant increase in SOCE and force generation in aortic rings in stoke-prone spontaneously hypertensive rats (SHRSP) compared to Wistar-Kyoto (WKY) controls [47]. In addition, SOCE-induced contraction was dramatically increased in mesenteric arteries from aged (22 months) compared to young (3 months) rats [46]. These data suggest SOCE plays a larger role in vascular function in diseased/aged states. Therefore, although we do not see a role for ASIC1a and Orai1 in basal SOCE responses in mesenteric arteries, these channels may exhibit a larger role in the enhanced SOCE response associated with cardiovascular diseases.

In summary, this study demonstrates the vast heterogeneity that exists between vascular beds. Whereas SOCE is very prominent in the pulmonary circulation and contributes to pulmonary vasoconstriction; in the mesenteric circulation the role of SOCE to vascular reactivity is negligible. Moreover, we have found that VSMC expression of ASIC1a plays a unique role in the pulmonary circulation to mediate vasoconstriction. As this study only investigated pulmonary versus mesenteric arteries, further investigation is warranted to determine the importance of SOCE and ASIC1a in other systemic vascular beds. In addition, it is important to determine whether SOCE is enhanced and becomes a fundamental signaling pathway that mediates the vascular dysfunction that occurs with many cardiovascular diseases. This knowledge, and the specific ion channels involved, will provide potential molecular targets to treat cardiovascular diseases.

## Supporting information

S1 Fig Primary pulmonary and mesenteric VSMC homogeneity is verified by morphological appearance and presence of smooth muscle marker. A: representative immunofluorescence images of pulmonary and mesenteric VSMC stained for smooth muscle 22α. B: representative PCR gels showing expression of smooth muscle α actin (F: 5’-ACTGCTGCTTCCTCTTCTTC-3’; R: 5’-GGCCAGCTTCGTCATACTCC-3’), calcitonin gene-related peptide (F: 5’-GTTCTCCCCTTTCCTGGTTG-3’; R: 5-’CTGGGGCTGTTATCTGTTCA-3’) and β-actin.
(PDF)

S2 Fig STIM1 is expressed in both pulmonary and mesenteric isolated arteries. Representative A: PCR gel showing mRNA expression of STIM1 in pulmonary (PA) and mesenteric (MA) arteries and brain (positive control; F: 5’-ATGCCAATGGTGATGTGGAT-3’; R: 5’-CCATGGAAGGTGCTGTGTTT-3’). B: Representative western blot showing protein expression in PA, MA, and brain (top; Abcam: ab108994), Coomassie blue was used to measure even loading (B-bottom).
(PDF)

S3 Fig STIM1, Orai1, and ASIC1 are expressed in both pulmonary and mesenteric VSMC. Representative PCR gel showing mRNA expression of STIM1, Orai1, and ASIC1 in both pulmonary and mesenteric primary cultured VSMC.
(PDF)

S4 Fig Negative control proximity ligation assay experiments. Representative images of negative control experiments where each primary antibody was incubated individually with subsequent incubation with probes.
(PDF)

S1 Raw images (PDF)
