# Supplementary material for: Coupling of store-operated calcium entry to vasoconstriction is acid-sensing ion channel 1a dependent in pulmonary but not mesenteric arteries
Source: PLoS One. 2020 Jul 23;15(7):e0236288. doi: 10.1371/journal.pone.0236288 (PMC7377459; doi:10.1371/journal.pone.0236288)
Supplement: S1 Fig — A: representative immunofluorescence images of pulmonary and mesenteric VSMC stained for smooth muscle 22α. B: representative PCR gels showing expression of smooth muscle α actin (F: 5’-ACTGCTGCTTCCTCTTCTTC-3’; R: 5’-GGCCAGCTTCGTCATACTCC-3’), calcitonin gene-related peptide (F: 5’-GTTCTCCCCTTTCCTGGTTG-3’; R: 5-’CTGGGGCTGTTATCTGTTCA-3’) and β-actin. (PDF) [file pone.0236288.s001.pdf]

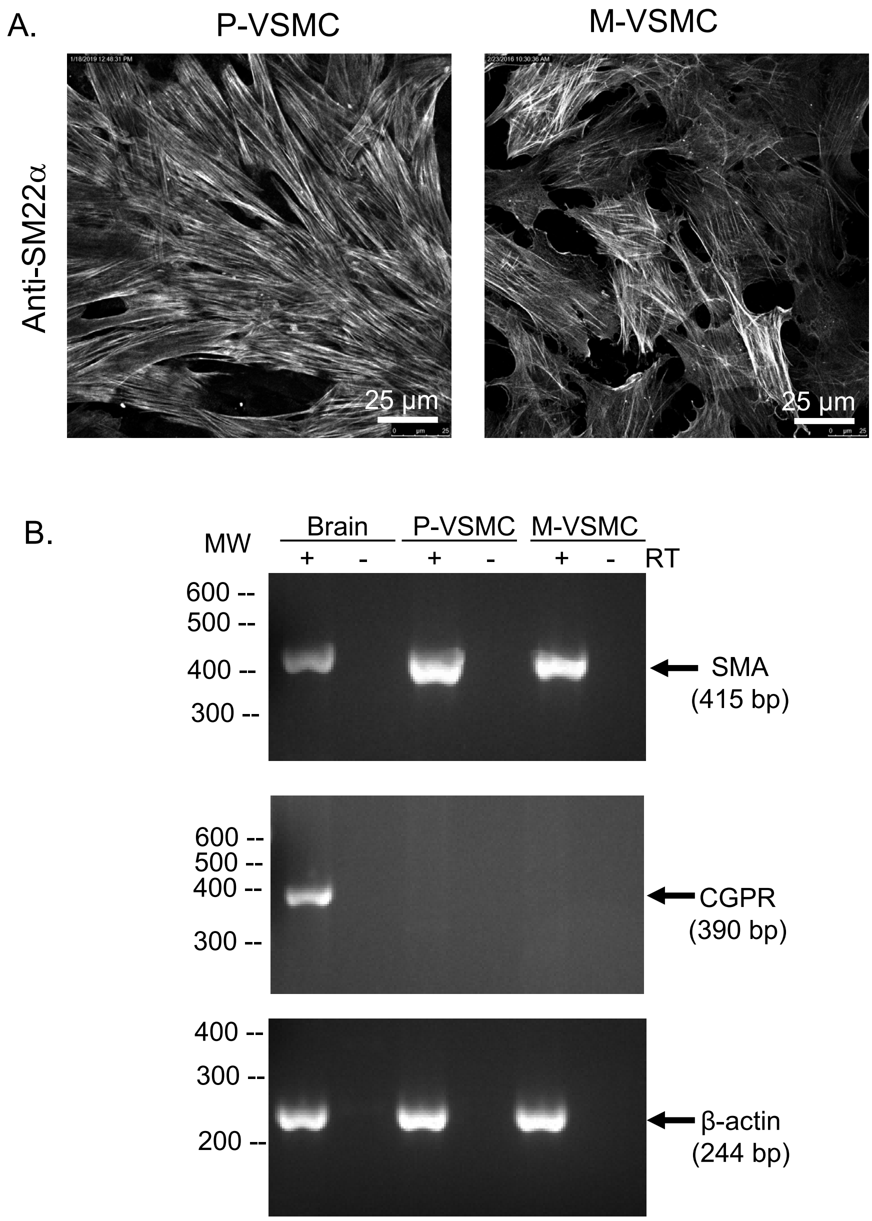

Figure S1: Primary pulmonary and mesenteric VSMC homogeneity is verified by morphological appearance and presence of smooth muscle marker. A: **r**epresentative immunofluorescence images of pulmonary and mesenteric VSMC stained for smooth muscle 22**α**. B: representative PCR gels showing expression of smooth muscle **α** actin (F: 5'-ACTGCTGCTTCCTCTTCTTC-3'; R: 5'-GGCCAGCTTCGTCATACTCC-3'), calcitonin gene-related peptide (F: 5'-GTTCTCCCCTTTCCTGGTTG-3'; R: 5-'CTGGGGCTGTTATCTGTTCA-3') and **β**-actin.
