# Supplementary material for: Coupling of store-operated calcium entry to vasoconstriction is acid-sensing ion channel 1a dependent in pulmonary but not mesenteric arteries
Source: PLoS One. 2020 Jul 23;15(7):e0236288. doi: 10.1371/journal.pone.0236288 (PMC7377459; doi:10.1371/journal.pone.0236288)
Supplement: S2 Fig — Representative A: PCR gel showing mRNA expression of STIM1 in pulmonary (PA) and mesenteric (MA) arteries and brain (positive control; F: 5’-ATGCCAATGGTGATGTGGAT-3’; R: 5’-CCATGGAAGGTGCTGTGTTT-3’). B: Representative western blot showing protein expression in PA, MA, and brain (top; Abcam: ab108994), Coomassie blue was used to measure even loading (B-bottom). (PDF) [file pone.0236288.s002.pdf]

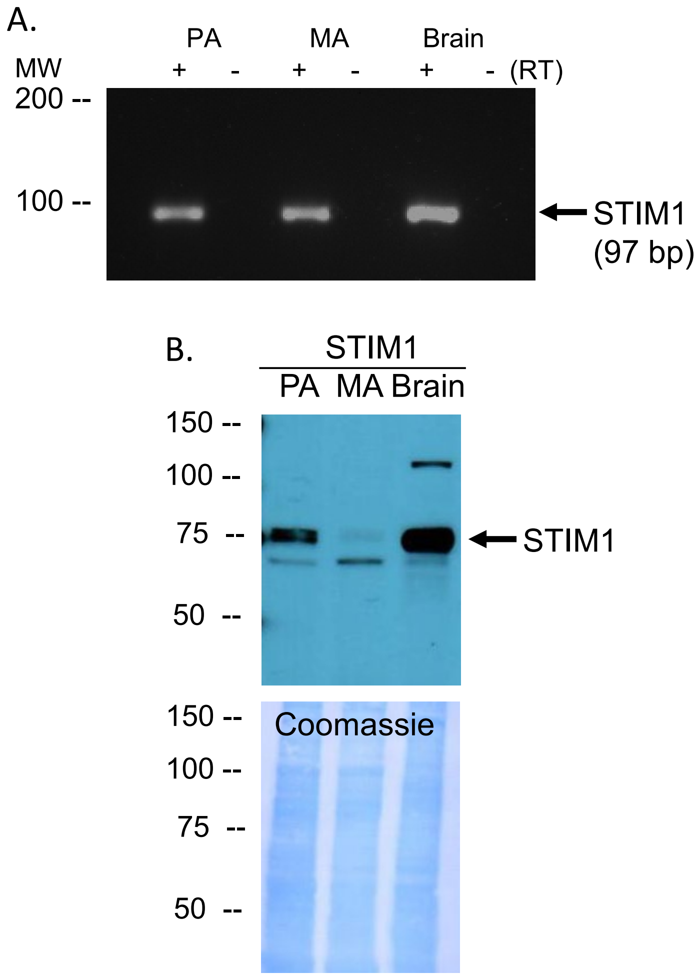

Figure S2: STIM1 is expressed in both pulmonary and mesenteric isolated arteries. Representative A: PCR gel showing mRNA expression of STIM1 in pulmonary (PA) and mesenteric (MA) arteries and brain (positive control; F: 5’-ATGCCAATGGTGATGTGGAT-3’; R: 5’-CCATGGAAGGTGCTGTGTTT-3’). B: Representative western blot showing protein expression in PA, MA and brain (top; Abcam: ab108994; expected MW 77 kDa), Coomassie blue was used to measure even loading (B-bottom).
