# Supplementary material for: Coupling of store-operated calcium entry to vasoconstriction is acid-sensing ion channel 1a dependent in pulmonary but not mesenteric arteries
Source: PLoS One. 2020 Jul 23;15(7):e0236288. doi: 10.1371/journal.pone.0236288 (PMC7377459; doi:10.1371/journal.pone.0236288)
Supplement: S3 Fig — Representative PCR gel showing mRNA expression of STIM1, Orai1, and ASIC1 in both pulmonary and mesenteric primary cultured VSMC. (PDF) [file pone.0236288.s003.pdf]

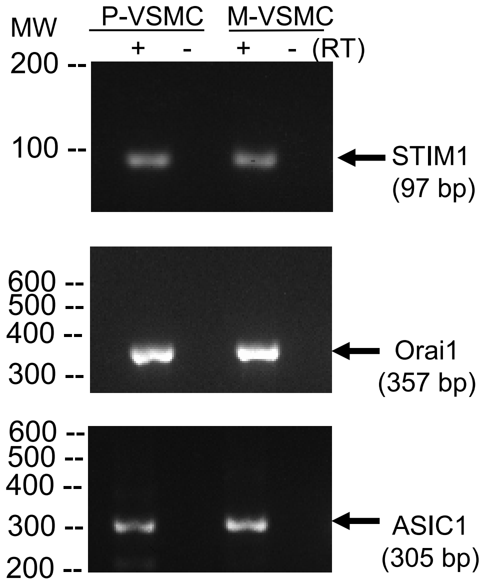

Figure S3: STIM1, Orai1 and ASIC1 are expressed in both pulmonary and mesenteric VSMC. Representative PCR gel showing mRNA expression of STIM1, Orai1 and ASIC1 in both pulmonary and mesenteric primary cultured VSMC.
