# Supplementary material for: Coupling of store-operated calcium entry to vasoconstriction is acid-sensing ion channel 1a dependent in pulmonary but not mesenteric arteries
Source: PLoS One. 2020 Jul 23;15(7):e0236288. doi: 10.1371/journal.pone.0236288 (PMC7377459; doi:10.1371/journal.pone.0236288)
Supplement: S4 Fig — Representative images of negative control experiments where each primary antibody was incubated individually with subsequent incubation with probes. (PDF) [file pone.0236288.s004.pdf]

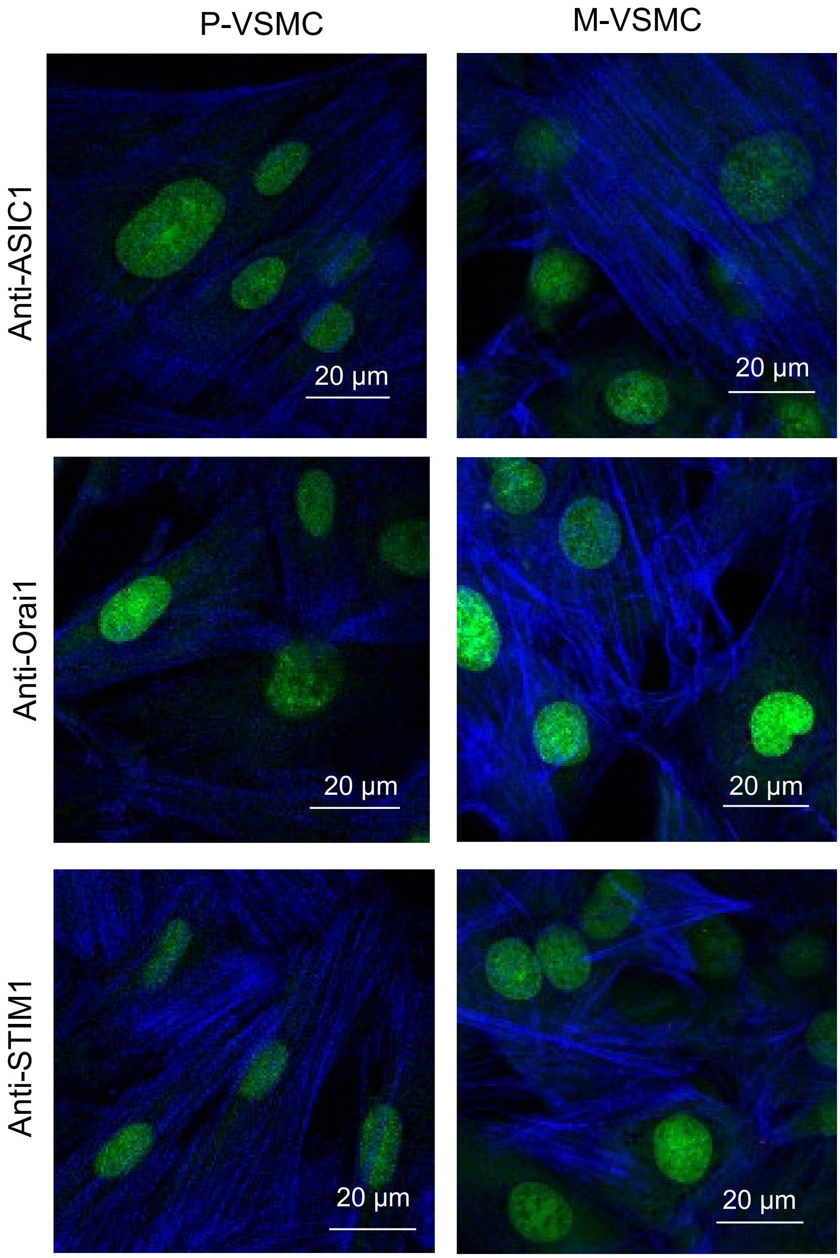

Figure S4: Negative control proximity ligation assay experiments. Representative images of negative control experiments where each primary antibody was incubated individually with subsequent incubation with probes.
