# Supplementary material for: Coupling of store-operated calcium entry to vasoconstriction is acid-sensing ion channel 1a dependent in pulmonary but not mesenteric arteries
Source: PLoS One. 2020 Jul 23;15(7):e0236288. doi: 10.1371/journal.pone.0236288 (PMC7377459; doi:10.1371/journal.pone.0236288)
Supplement: S1 Raw images — (PDF) [file pone.0236288.s005.pdf]

Raw gel images from Figure 2A

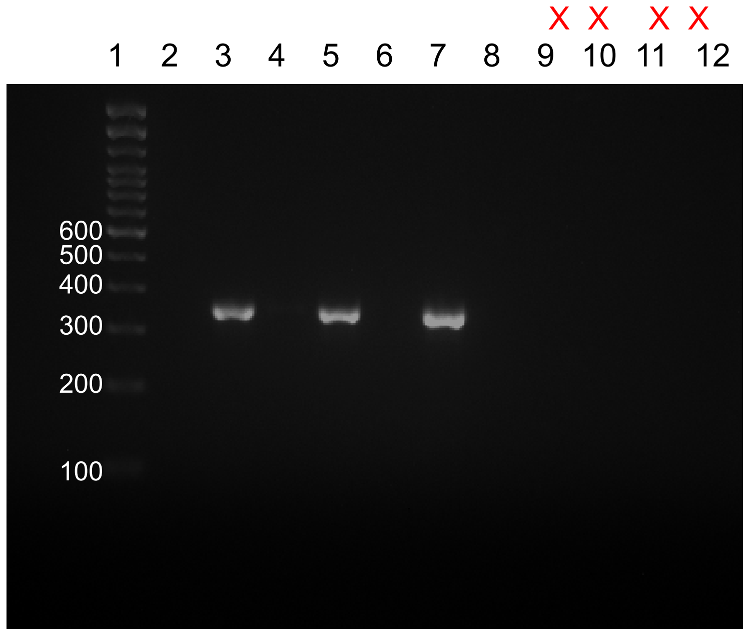

Orai1

Orai1<br>Primer Pair Sequence	Product Size, bp
F: 5’-ACGTCCACAACCTCAACTCC-3’<br>R: 5’-ACTGTCGGTCCGTCTTATGG-3’	357 bp

PCR products were separated using gel electrophoresis on a 3% agarose gel and stained with ethidium bromide for visualization under UV light.

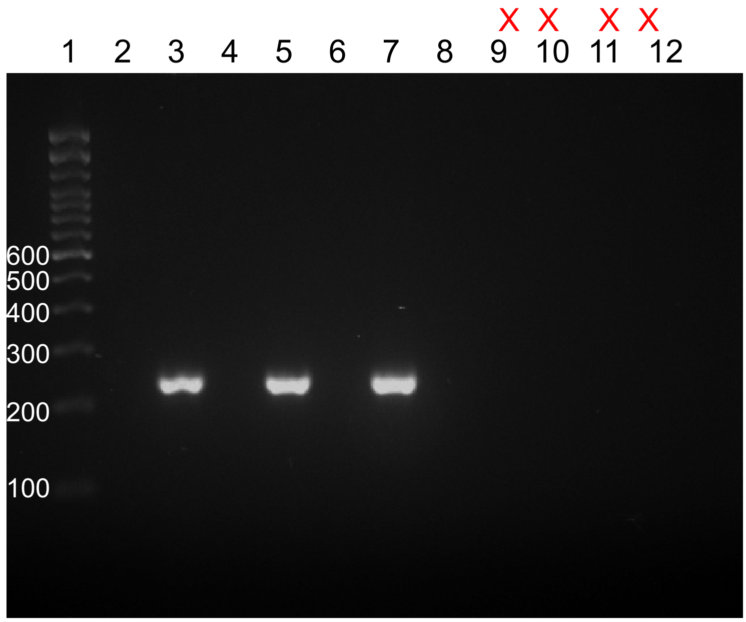

β-actin

**β**-actin<br>Primer Pair Sequence	Product Size, bp
F: 5’-AGTGTGACGTTGACATCCGT-3’<br>R: 5’-GACTCATCGTACTCCTGCTT-3’	244 bp

PCR products were separated using gel electrophoresis on a 3% agarose gel and stained with ethidium bromide for visualization under UV light.

1. Molecular weight marker
2. Blank
3. Pulmonary artery + reverse transcriptase
4. Pulmonary artery - reverse transcriptase
5. Mesenteric artery + reverse transcriptase
6. Mesenteric artery - reverse transcriptase
7. Brain tissue + reverse transcriptase
8. Brain tissue - reverse transcriptase
9. Blank
10. Blank
11. Blank
12. Blank

Raw gel images from Figure 2A

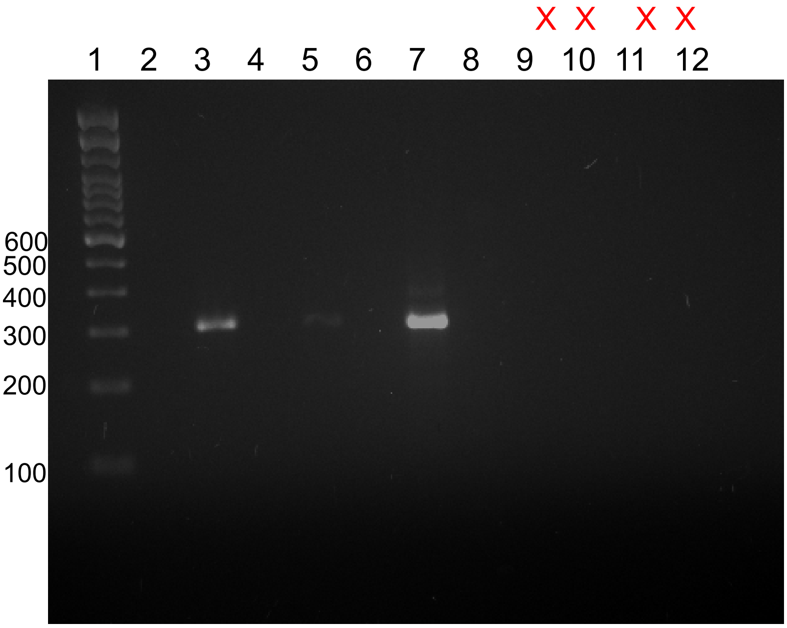

ASIC1: Even Loading

ASIC1 Primer Pair Sequence	Product Size, bp
F: 5'-GCCTATGAGATCGCAGGG-3'<br>R: 5'-AAAGTCCTCAAACGTGCCTC-3'	305 bp

PCR products were separated using gel electrophoresis on a 3% agarose gel and stained with ethidium bromide for visualization under UV light. ASIC1 transcript Fig. 2b (even loading).

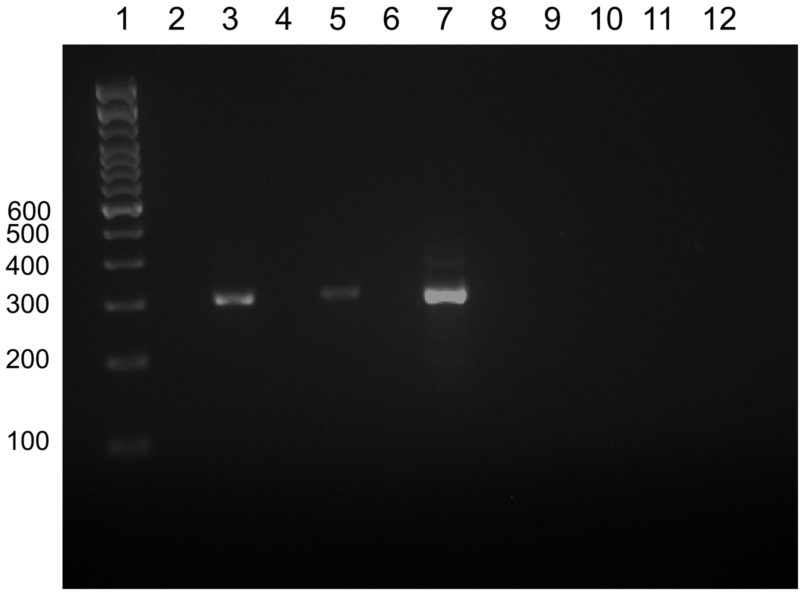

Uneven Loading
(Gel shown for reviewers, not shown in manuscript)

Uneven loading of ASIC1 transcript from above. Pulmonary and brain samples were loaded with 5μl cDNA while mesenteric was loaded with 15μl cDNA. Shown here to help visualize mesenteric band.

1. Molecular weight marker
2. Blank
3. Pulmonary artery + reverse transcriptase
4. Pulmonary artery - reverse transcriptase
5. Mesenteric artery + reverse transcriptase
6. Mesenteric artery - reverse transcriptase
7. Brain tissue + reverse transcriptase
8. Brain tissue - reverse transcriptase
9. Blank
10. Blank
11. Blank
12. Blank

Raw blot images from Figure 2B –ORAI1

Anti-Orai1 (Proteintech #14443-1-AP) 1.5 hrs @ 1:300; expected MW 35-44 kDa

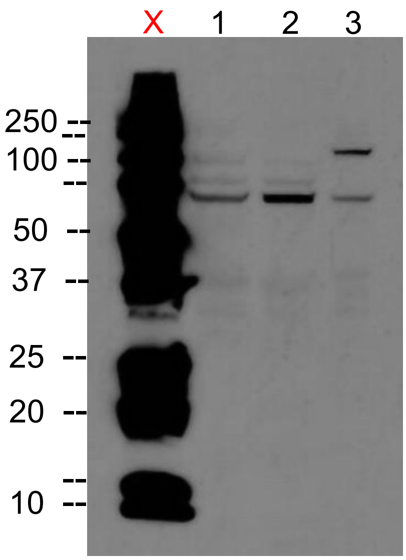

Exposure Time: 3 minutes

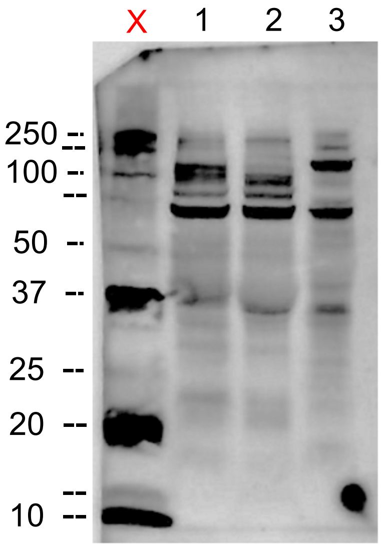

Exposure Time: 3 hrs

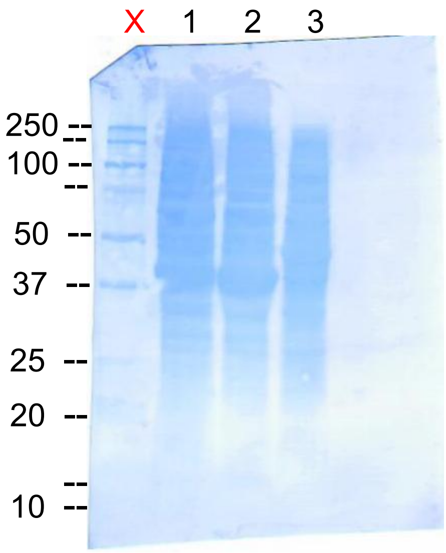

Coomassie

Anti-Orai1 (Alomone #ACC-0621:100; overnight @ 1:100; expected MW not reported)

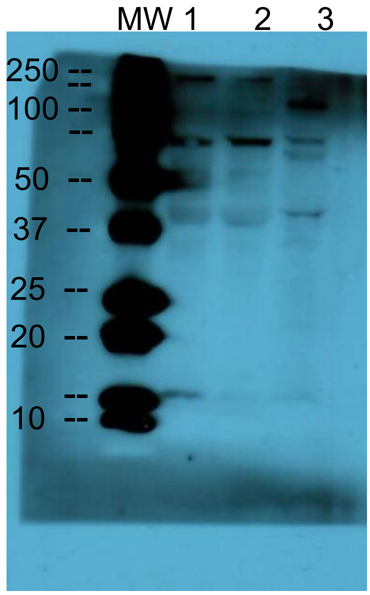

Exposure Time: 1 minutes

(Gel shown for reviewers, not shown in manuscript)

1. Pulmonary artery
2. Mesenteric artery
3. Brain tissue

Western blots were incubated with -horseradish peroxidase, followed by chemiluminescence labeling. Proteins were detected by exposing the blot to chemiluminescence-sensitive film. Blots were then stained with Coomassie blue to use as loading control.

Raw western blot images from Figure 2B – ASIC1

Anti-ASIC1 (Millipore: AB5674P) 48 hrs @ 1:500; reported MW ~60 and ~100 kDa

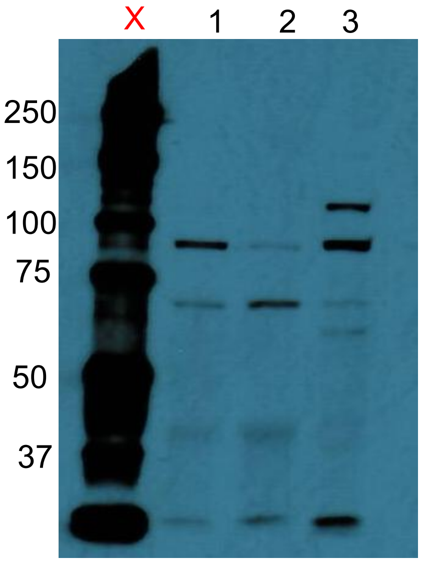

Exposure: 3 minute

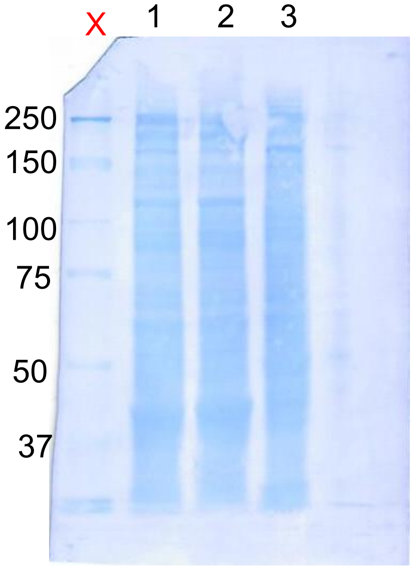

Coomassie

1. Pulmonary artery
2. Mesenteric artery
3. Brain tissue

Western blots were incubated with -horseradish peroxidase, followed by chemiluminescence labeling. Proteins were detected by exposing the blot to chemiluminescence-sensitive film. Blots were then stained with Coomassie blue to use as loading control.

Raw gel images from Figure S1B

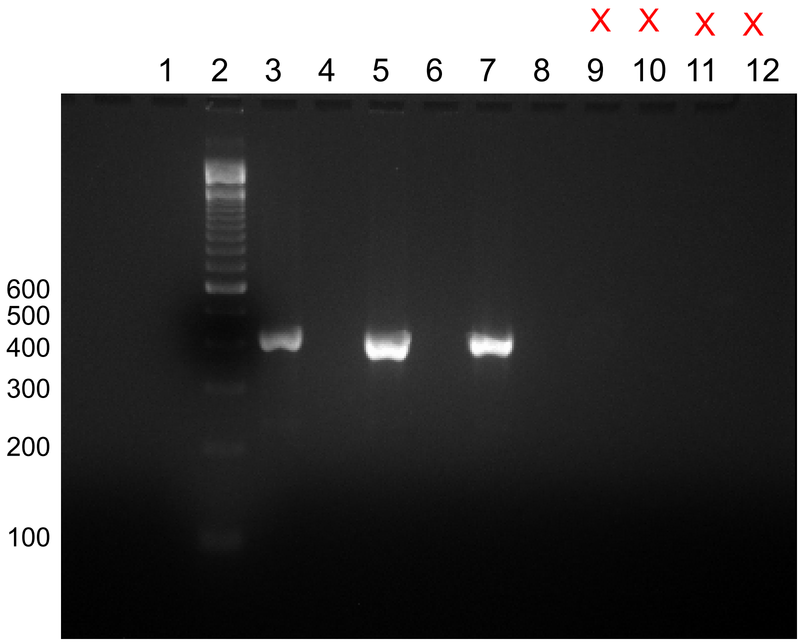

Smooth Muscle α-actin (SMA

SMA<br>Primer Pair Sequence	Product Size, bp
F: 5’-ACTGCTGCTTCCTCTTCTTC-3’<br>R: 5’-GGCCAGCTTCGTCATACTCC-3’	415 bp

PCR products were separated using gel electrophoresis on a 3% agarose gel and stained with ethidium bromide for visualization under UV light.

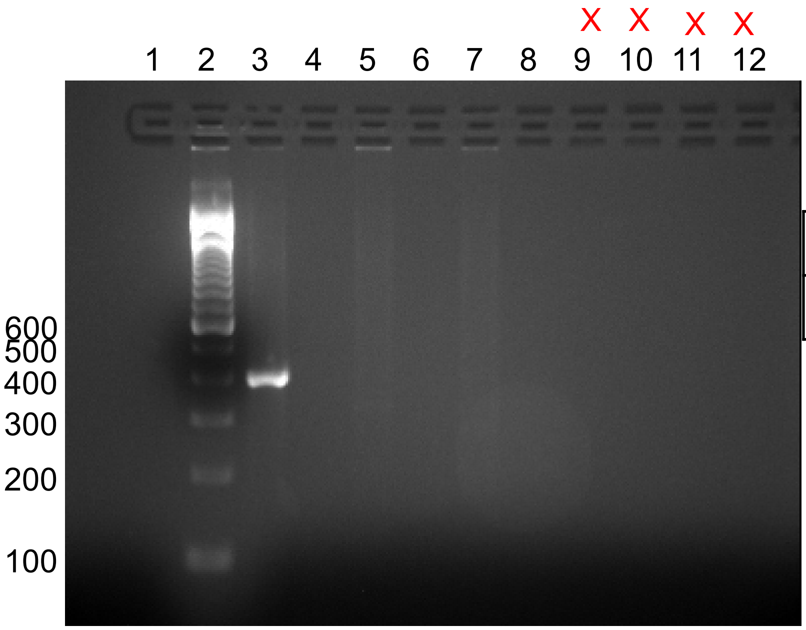

calcitonin gene-related peptide (CGRP)

CGRP<br>Primer Pair Sequence	Product Size, bp
F: 5’-TTCTCCCCTTTCCTGGTTG-3’<br>R: 5-CTGGGGCTGTTATCTGTTCA-3’	390 bp

PCR products were separated using gel electrophoresis on a 3% agarose gel and stained with ethidium bromide for visualization under UV light.

1. Blank
2. Molecular weight marker
3. Brain tissue + reverse transcriptase
4. Brain tissue - reverse transcriptase
5. Pulmonary VSMC + reverse transcriptase
6. Pulmonary VSMC - reverse transcriptase
7. Mesenteric VSMC+ reverse transcriptase
8. Mesenteric VSMC- reverse transcriptase
9. Blank
10. Blank
11. Blank
12. Blank

Raw gel image from Figure S1B

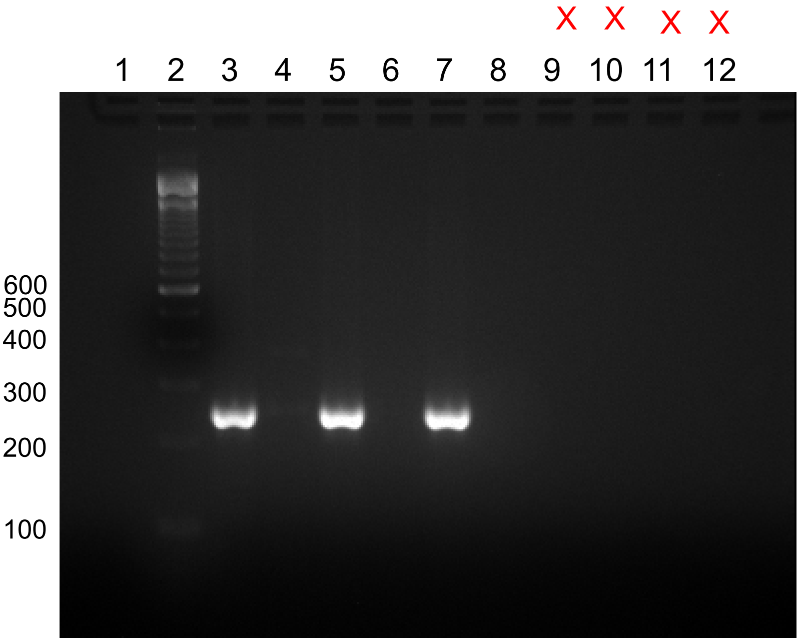

β-actin

β-actin<br>Primer Pair Sequence	Product Size, bp
F: 5’-AGTGTGACGTTGACATCCGT-3’<br>R: 5’-GACTCATCGTACTCCTGCTT-3’	244 bp

PCR products were separated using gel electrophoresis on a 3% agarose gel and stained with ethidium bromide for visualization under UV light. β-actin transcript Fig. S1B.

1. Blank
2. Molecular weight marker
3. Brain tissue + reverse transcriptase
4. Brain tissue - reverse transcriptase
5. Pulmonary VSMC + reverse transcriptase
6. Pulmonary VSMC - reverse transcriptase
7. Mesenteric VSMC+ reverse transcriptase
8. Mesenteric VSMC- reverse transcriptase
9. Blank
10. Blank
11. Blank
12. Blank

Raw gel and blot images from Figure S2

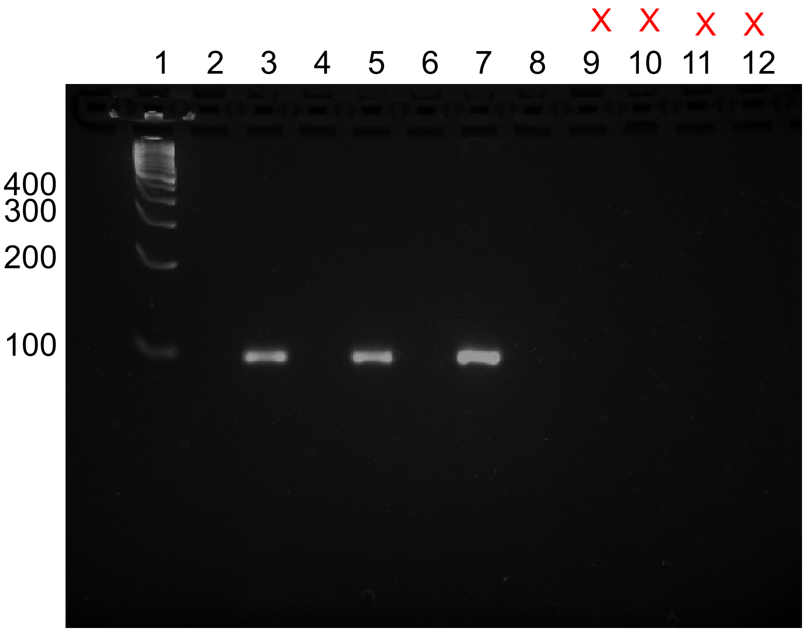

PCR products were separated using gel electrophoresis on a 3% agarose gel and stained with ethidium bromide for visualization under UV light.

Stromal Interaction Molecule 1

STIM1 Primer Pair Sequence	Product Size, bp
F: 5'-ATGCCAATGGTGATGTGGAT-3'<br>R: 5'-CATGGAAGGTGCTGTGTTT-3'	97 bp

1. Molecular weight marker
2. Blank
3. Pulmonary artery + reverse transcriptase
4. Pulmonary artery - reverse transcriptase
5. Mesenteric artery + reverse transcriptase
6. Mesenteric artery - reverse transcriptase
7. Brain tissue + reverse transcriptase
8. Brain tissue - reverse transcriptase
9. Blank
10. Blank
11. Blank
12. Blank

Rabbit anti-STIM1 (Abcam #ab108994, 1:500 overnight; predicted MW 77 kDa

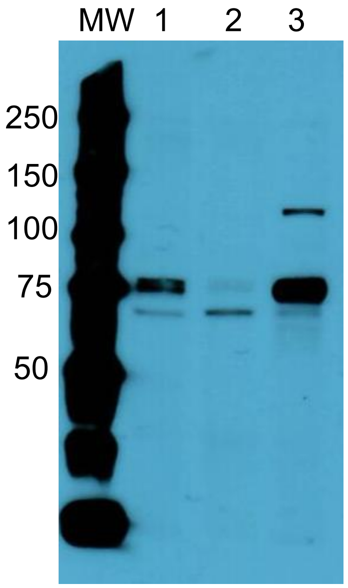

Exposure time: 1 minute

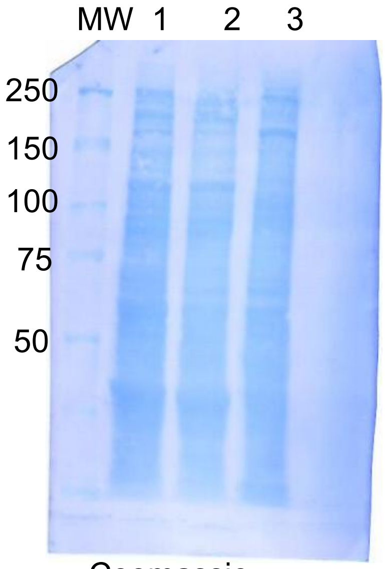

Coomassie

1. Pulmonary artery
2. Mesenteric artery
3. Brain tissue

Western blots were incubated with - horseradish peroxidase, followed by chemiluminescence labeling. Proteins were detected by exposing the blot to chemiluminescence-sensitive film. Blots were then stained with Coomassie blue to use as loading control.

Raw gel images from Figure S3

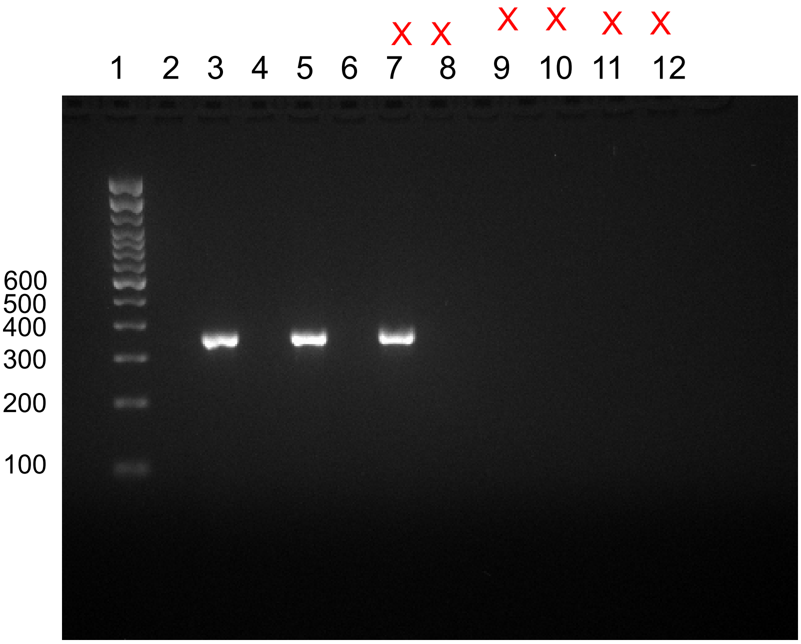

Orai1

Orai1 Primer Pair Sequence	Product Size, bp
F: 5'-ACGTCCACAACCTCAACTCC-3'<br>R: 5'-ACTGTCGGTCCGTCTTATGG-3'	357 bp

PCR products were separated using gel electrophoresis on a 3% agarose gel and stained with ethidium bromide for visualization under UV light.

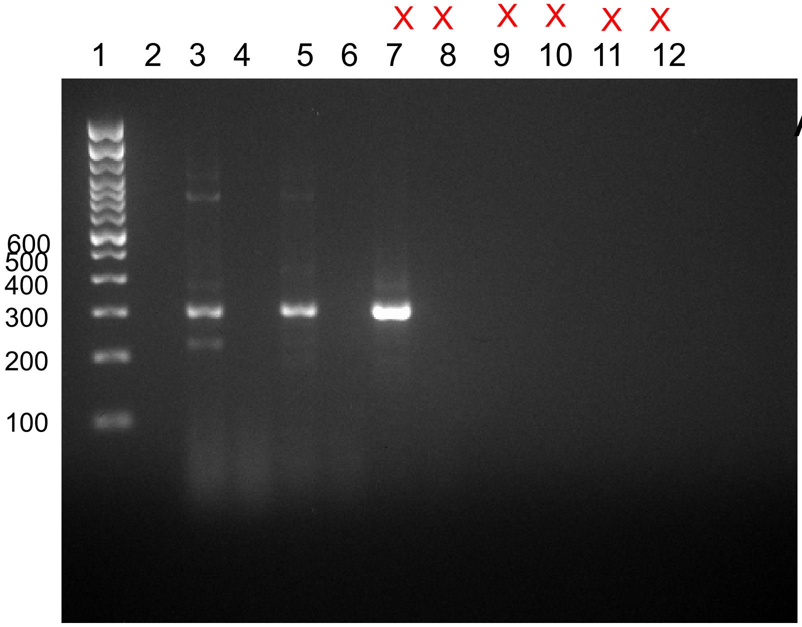

ASIC1

ASIC1 Primer Pair Sequence	Product Size, bp
F: 5'-GCCTATGAGATCGCAGGG-3'<br>R: 5'-AAAGTCCTCAAACGTGCCTC-3'	305 bp

PCR products were separated using gel electrophoresis on a 3% agarose gel and stained with ethidium bromide for visualization under UV light.

1. Molecular weight marker
2. Blank
3. Pulmonary VSMC + reverse transcriptase
4. Pulmonary VSMC - reverse transcriptase
5. Mesenteric VSMC + reverse transcriptase
6. Mesenteric VSMC - reverse transcriptase
7. Brain tissue + reverse transcriptase
8. Brain tissue - reverse transcriptase
9. Blank
10. Blank
11. Blank
12. Blank

Raw gel images from Figure S3

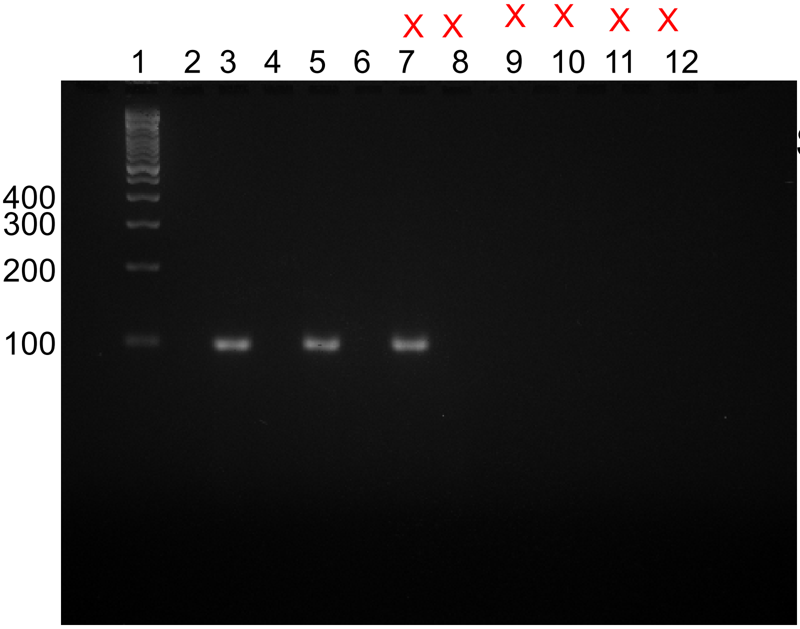

Stromal Interaction Molecule 1

STIM1<br>Primer Pair Sequence	Product Size, bp
F: 5’-ATGCCAATGGTGATGTGGAT-3’<br>R: 5’-CATGGAAGGTGCTGTGTTT-3’	97 bp

PCR products were separated using gel electrophoresis on a 3% agarose gel and stained with ethidium bromide for visualization under UV light. STIM1 transcript Fig. S3.

1. Molecular weight marker
2. Blank
3. Pulmonary VSMC + reverse transcriptase
4. Pulmonary VSMC - reverse transcriptase
5. Mesenteric VSMC + reverse transcriptase
6. Mesenteric VSMC - reverse transcriptase
7. Brain tissue + reverse transcriptase
8. Brain tissue - reverse transcriptase
9. Blank
10. Blank
11. Blank
12. Blank
